# Handling Several Sugars at a Time: a Case Study of Xyloglucan Utilization by *Ruminiclostridium cellulolyticum*

**DOI:** 10.1128/mBio.02206-21

**Published:** 2021-11-09

**Authors:** Clara Kampik, Nian Liu, Mohamed Mroueh, Nathalie Franche, Romain Borne, Yann Denis, Séverine Gagnot, Chantal Tardif, Sandrine Pagès, Stéphanie Perret, Nicolas Vita, Pascale de Philip, Henri-Pierre Fierobe

**Affiliations:** a Aix-Marseille Université, CNRS, LCB-UMR7283, Marseille, France; University of Delaware

**Keywords:** hemicellulose, xyloglucan, central carbon metabolism, *Ruminiclostridium cellulolyticum*, metabolic enzymes, simultaneous catabolism

## Abstract

Xyloglucan utilization by *Ruminiclostridium cellulolyticum* was formerly shown to imply the uptake of large xylogluco-oligosaccharides, followed by cytosolic depolymerization into glucose, galactose, xylose, and cellobiose. This raises the question of how the anaerobic bacterium manages the simultaneous presence of multiple sugars. Using genetic and biochemical approaches targeting the corresponding metabolic pathways, we observed that, surprisingly, all sugars are catabolized, collectively, but glucose consumption is prioritized. Most selected enzymes display unusual features, especially the GTP-dependent hexokinase of glycolysis, which appeared reversible and crucial for xyloglucan utilization. In contrast, mutant strains lacking either galactokinase, cellobiose-phosphorylase, or xylulokinase still catabolize xyloglucan but display variably altered growth. Furthermore, the xylogluco-oligosaccharide depolymerization process appeared connected to the downstream pathways through an intricate network of competitive and noncompetitive inhibitions. Altogether, our data indicate that xyloglucan utilization by *R. cellulolyticum* relies on an energy-saving central carbon metabolism deviating from current bacterial models, which efficiently prevents carbon overflow.

## INTRODUCTION

The diauxic shift, which was demonstrated for a number of bacteria, including the model prokaryotes Escherichia coli (1) and Bacillus subtilis (2), implies that when two (or more) carbohydrates are available in the environment, the microorganisms utilize one sugar preferentially, with the remaining carbohydrate(s) being catabolized only when the preferred one is almost totally depleted. The diauxic shift observed for carbohydrates relies on carbon catabolite repression, which often includes phosphotransferase systems, for substrate import. Some bacteria can coutilize a saccharide and another carbon source entering the metabolic network at different/downstream points such as succinate or acetate (3, 4), but only few reports describe the coutilization of two sugars by bacteria (5, 6).

Nevertheless, this scheme cannot apply to (hemi)cellulolytic bacteria whose selected strategy to utilize plant cell wall polysaccharides involves the uptake of rather large oligosaccharides (7, 8), often composed of different monosaccharides (9–13). Once their cytosolic depolymerization is achieved, the bacterium faces the simultaneous intracellular release of different sugars requiring different metabolic pathways. In this respect, the catabolism of xyloglucan by the anaerobic Gram-positive bacterium Ruminiclostridium cellulolyticum is of particular interest, since the utilization of this complex polysaccharide was formerly shown to imply the cytosolic release of glucose, xylose, galactose, and the disaccharide cellobiose (9). In this particular context, exploring the coordination of the various downstream metabolic pathways in *R. cellulolyticum* could shed new light on how a bacterial carbon central metabolism can function without relying on the diauxic shift. *R. cellulolyticum* and related clostridia are important players of the terrestrial carbon cycle and occupy various anaerobic biotopes where plant cell wall polymers such as xyloglucan accumulate. The latter is a prominent plant cell wall polysaccharide (14, 15), composed of a main chain of β-1,4-linked glucosyl residues (designated G) carrying various decorations forming regular patterns. For instance, in dicot species such as tamarind, xyloglucan exhibits a series of three glucosyl residues decorated with α-1,6 xylosyl residues (designated X), followed by a single undecorated glucosyl residue (G), thus leading to the motif XXXG. The second and/or third xylosyl residues can in turn be decorated with a β-1,2 galactosyl residue (L), thereby leading to the motifs XLXG, XXLG, or XLLG (16).

**FIG 1:**
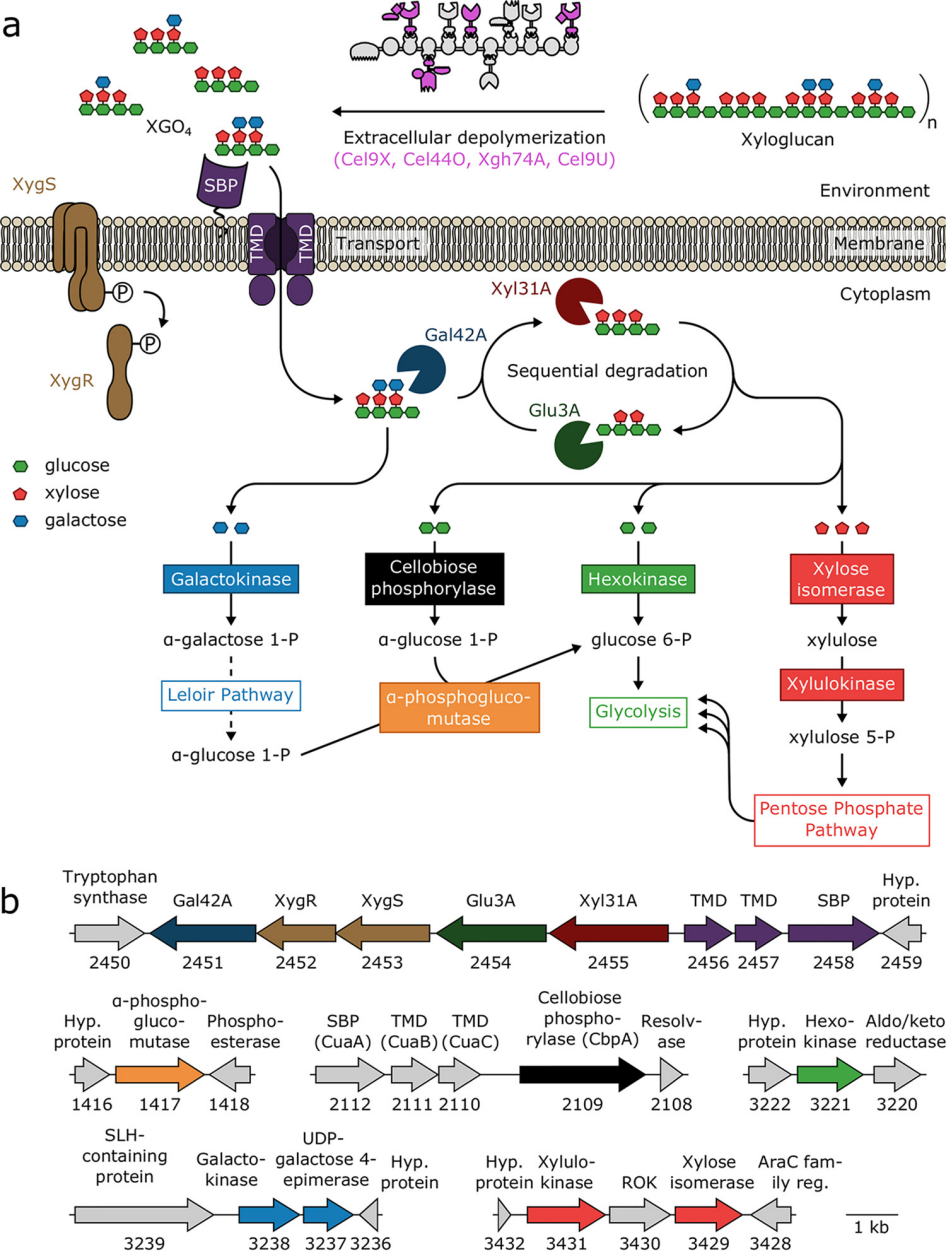
Xyloglucan degradation by *R. cellulolyticum* and relevant genes. (a) Schematic representation of xyloglucan degradation by *R. cellulolyticum* and corresponding downstream metabolic pathways. Xyloglucan is converted into xyloglucan oligosaccharides (XGO_4_) by cellulosomes (gray) containing the identified cellulosomal enzymes Cel9X, Cel44O, Xgh74A, and Cel9U (pink) active on xyloglucan. The oligosaccharides are subsequently imported into the cell through a specific ABC-transporter (purple). The next step involves the sequential, intracellular degradation of the imported XGO_4_ by the β-galactosidase Gal42A (dark blue partial circle), the α-xylosidase Xyl31A (dark red partial circle), and the β-glucosidase Glu3A (dark green partial circle) into galactose (blue diamond), xylose (red diamond), glucose (green diamond), and cellobiose. The XygS/XygR two-component system (brown) is involved in the positive transcriptional regulation of the genes encoding the three intracellular degradation enzymes, the ABC transporter, and the two-component system itself. The metabolic enzymes targeted in the present study and the corresponding pathways are shown in boxes. This scheme was adapted from a previous study (9). (b) Genetic context of relevant/targeted genes. The genes are designated by arrows. The predicted encoded proteins and gene loci (e.g., Ccel_2450) are indicated above and below each gene, respectively. TMD and SBP designate the TransMembrane Domain and Solute Binding Protein of ABC-transporters. Hyp. protein indicates hypothetical protein, reg. indicates regulator, ROK indicates Repressor Orf Kinase, and SLH indicates the S-Layer Homologous domain. CuaA/B/C (Ccel_2112-2110) were formerly characterized as a cellodextrin ABC-transporter (22).

The utilization of tamarind xyloglucan in *R. cellulolyticum* is based on the extracellular depolymerization of xyloglucan by multienzymatic complexes called cellulosomes. Four cellulosomal enzymes—Cel9U, Cel9X, Cel44O, and Xgh74A—were previously shown to exhibit elevated activities on xyloglucan and generate 4-glucosyl-backbone dextrins (Fig. 1a) (9, 17). The latter are subsequently imported by a specific ABC-transporter into the cytosol, where their depolymerization into galactose, glucose, xylose, and the disaccharide cellobiose is achieved by a specific β-galactosidase, α-xylosidase, and β-glucosidase acting sequentially (9) (Fig. 1a). The expression of the genes encoding the cytosolic enzymes and the ABC-transporter was formerly shown to be strongly induced by xyloglucan (9, 18).

Other examples related to the simultaneous presence of multiple sugars in the cytosolic compartment of prokaryotes have, to our knowledge, not been addressed, and the question arises how *R. cellulolyticum* manages the concurrent release of glucose, xylose, galactose, and cellobiose? To address this fundamental question, key enzymes of each required metabolic pathway and their encoding genes were targeted using a combination of biochemical and genetic approaches. Our data show that, unexpectedly, *R. cellulolyticum* catabolizes the four released sugars collectively, but at different velocities, and through unconventional metabolic enzymes. Our data also demonstrate that the upstream cytoplasmic depolymerization of xylogluco-oligosaccharides is tightly connected to the downstream pathways and efficiently prevents carbon overflow, i.e., the saturation of the required metabolic pathways.

## RESULTS

### Selection of targeted genes and enzymes.

To investigate how *R. cellulolyticum* catabolizes the four sugars released by the cytosolic depolymerization of xylogluco-oligosaccharides, we initially targeted the first enzymes involved in each required pathway. Thus, the predicted hexokinase (19) from glycolysis, the predicted galactokinase (20) from the Leloir pathway, and the predicted xylose isomerase (21) that drives xylose to the pentose phosphate pathway (PPP) were examined (Fig. 1a). With respect to cellobiose, a cellobiose phosphorylase named CbpA, encoded by the gene at locus Ccel_2109, was formerly purified and shown to be essential for the growth of *R. cellulolyticum* on cellobiose-based medium (Fig. 1) (22). This enzyme was therefore included in the present study. Analysis of the genome suggested that the genes at loci Ccel_3221 and Ccel_3238 encode the sole hexokinase and galactokinase, respectively (Fig. 1b). Regarding the catabolism of xylose, four genes (at loci Ccel_0500, Ccel_0941, Ccel_1925, and Ccel_3429) potentially encode a xylose isomerase, but the gene at locus Ccel_0941 was shown to encode a d-psicose isomerase (23). The gene at locus Ccel_3429 is located one gene downstream of the only gene in *R. cellulolyticum* (locus Ccel_3431) predicted to encode a xylulokinase (Fig. 1b), which converts the product of the xylose isomerase into xylulose 5-P, which then enters PPP (Fig. 1a). This gene was therefore assumed to encode the major xylose isomerase and selected with its product for genetic and biochemical approaches. Finally, the gene at locus Ccel_1417 and its product, the α-phosphoglucomutase (24) (Fig. 1a and b), were also investigated. Though this enzyme is not the first enzyme of a pathway, it was expected to play a major role in xyloglucan catabolism, since it converts α-d-glucose 1-phosphate (Glu1P), produced by both CbpA and the Leloir pathway (Fig. 1), into d-glucose 6-phosphate (Glu6P), which enters the glycolytic pathway.

The expression pattern of the selected genes was explored by RT-qPCR, using RNA extracted from cultures of *R. cellulolyticum* grown on arabinose- and xyloglucan-based medium. Only small positive changes of the relative expression levels were detected for the xyloglucan-grown cells, except for the gene encoding the galactokinase (see Fig. S1 in the supplemental material), for which a 10-fold increased expression was observed on xyloglucan.

10.1128/mBio.02206-21.3 FIG S1 Relative expression of genes encoding the selected metabolic enzymes in *R. cellulolyticum*. The cultures used for RNA preparation were grown on arabinose (white) or xyloglucan (blue). They were inoculated from arabinose-containing precultures. For each gene, the relative expression on a given growth substrate is standardized with 16S rRNA encoding gene amplification and given versus arabinose. Error bars show the standard errors of three biological replicates. RNA extraction, reverse transcription, and quantitative real-time PCR (qPCR) analyses were performed as formerly described (18). Briefly, total RNAs were prepared from *R. cellulolyticum* 20-ml cultures grown until late exponential phase, and the RNA was quantified and reverse transcribed. Quantitative real-time PCR (RT-qPCR) analyses were performed on a CFX96 real-time system (Bio-Rad), and the results were analyzed using Bio-Rad CFX Maestro software, version 1.1 (Bio-Rad, France) (18). The RNA16S gene was used as a reference for normalization. Primer pairs are reported in Table S2. Gene loci and corresponding enzymes are given below each graph. Download FIG S1, DOCX file, 0.06 MB. Copyright © 2021 Kampik et al. 2021 Kampik et al. https://creativecommons.org/licenses/by/4.0/ This content is distributed under the terms of the Creative Commons Attribution 4.0 International license.

10.1128/mBio.02206-21.2 TABLE S2 Primers used in this study. Download Table S2, DOCX file, 0.02 MB. Copyright © 2021 Kampik et al. 2021 Kampik et al. https://creativecommons.org/licenses/by/4.0/ This content is distributed under the terms of the Creative Commons Attribution 4.0 International license.

### Enzyme characterization and gene inactivation.

All enzymes mentioned above were grafted to a C-terminal 6×His tag, overproduced in E. coli, and purified to homogeneity. Their kinetic parameters (Table 1) were determined at pH 7.0 and 37°C.

**TABLE 1:** Kinetic parameters of the selected metabolic enzymes

Enzyme	Substrate(s) (fixed cosubstrate concn)^*a*^			Inhibitor
Cellobiose phosphorylase CbpA	Cellobiose *k*_cat_ = 1,458 ± 10 *K_m_* = 2.85 ± 0.47*k*_cat_/*K_m_* = 511.6 ± 0.47			Glucose *K_i_* = 0.68 ± 0.04^*b*^
Hexokinase	Glucose (ATP, 25 mM)^*c*^ *k*_cat_ = 3,341 ± 60 *K_m_* = 0.17 ± 0.015 *k*_cat_/*K_m_* = 19,653 ± 1,769	ATP (glucose, 5 mM)^*d*^ *k*_cat_ = 8,781 ± 1,418 *K_m_* = 32.2 ± 7.4 *k*_cat_/*K_m_* = 272.7 ± 77	Mannose (ATP, 25 mM) *k*_cat_ = 3,610 ± 151 *K_m_* = 0.27 ± 0.05 *k*_cat_/*K_m_* = 13,370 ± 2,538	None^*h*^
				
	Glucose (GTP, 2 mM)^*e*^ *k*_cat_ = 8,804 ± 177 *K_m_* = 0.21 ± 0.017 *k*_cat_/*K_m_* = 41,924 ± 3,497	GTP (glucose, 5 mM) *k*_cat_ = 11,160 ± 543 *K_m_* = 0.40 ± 0.07 *k*_cat_/*K_m_* = 27,900 ± 5,067	Mannose (GTP, 2 mM) *k*_cat_ = 5,569 ± 79 *K_m_* = 0.12 ± 0.01 *k*_cat_/*K_m_* = 46,408 ± 3,923	
Galactokinase	Galactose (ATP, 25 mM) *k*_cat_ = 3,771 ± 128 *K_m_* = 20.55 ± 2.18 *k*_cat_/*K_m_* = 183.5 ± 20.4	ATP (galactose, 100 mM)^*f*^ *k*_cat_ = 4,853 ± 419 *K_m_* = 13.71 ± 2.42 *k*_cat_/*K_m_* = 354 ± 69.6		None^*i*^
				
	Galactose (GTP, 5 mM)^*g*^ *k*_cat_ = 1,815 ± 62 *K_m_* = 15.5 ± 1.8 *k*_cat_/*K_m_* = 117.1 ± 14.2	GTP (galactose, 100 mM) *k*_cat_ = 1,704 ± 75 *K_m_* = 0.99 ± 0.13 *k*_cat_/*K_m_* = 1,721 ± 238		
Xylose isomerase	Xylose *k*_cat_ = 103.2 ± 3.9 *K_m_* = 23.85 ± 2.38 *k*_cat_/*K_m_* = 4.33 ± 0.46	Xylulose *k*_cat_ = 63. 5 ± 1.8 *K_m_* = 2.0 ± 0.2 *k*_cat_/*K_m_* = 31.8 ± 3.3		None^*j*^
α-Phosphoglucomutase	Glu1P *k*_cat_ = 1,768 ± 19 *K_m_* = 0.155 ± 0.055 *k*_cat_/*K_m_* = 11,406 ± 4,049	Glu6P *k*_cat_ = 1,135 ± 55 *K_m_* = 3.87 ± 0.33 *k*_cat_/*K_m_* = 293.3 ± 28.8		ND

a *k*_cat_ values are given in IU μmol^−1^. The data show the means and standard deviations of two or three replicates. *K_m_* and *K_i_* values are given in mM. The data show the means and standard deviations of two or three replicates. *k*_cat_/*K_m_* values are given IU μmol^−1^ mM^−1^. ND, not determined.

b Competitive inhibition.

c The kinetic parameters were determined in the presence of 25 mM ATP.

d The kinetic parameters were determined in the presence of 5 mM glucose.

e The kinetic parameters were determined in the presence of 2 mM GTP.

f The kinetic parameters were determined in the presence of 100 mM galactose.

g The kinetic parameters were determined in the presence of 5 mM GTP.

h No inhibition observed in the presence of 20 mM cellobiose, 20 mM galactose, or 20 mM xylose.

i No inhibition observed in the presence of 20 mM cellobiose, 20 mM glucose, or 20 mM xylose.

j No inhibition observed in the presence of 20 mM cellobiose, 20 mM galactose, or 20 mM glucose.

The cellobiose phosphorylase CbpA catalyzes the phosphorolysis of cellobiose into glucose and Glu1P. Its kinetic parameters (Table 1) were formerly determined (22). Since the various metabolic pathways considered in this study may be interconnected through cross inhibitions/activations, the CbpA activity was also assayed in the presence of various sugars. Xylose and galactose at 20 mM did not induce any alteration of its activity, whereas glucose is a potent competitive inhibitor with a *K*_I_ value of 0.68 mM (Table 1; see also Fig. S2e). Inactivation of the corresponding gene, using the ClosTron technology (25), was formerly shown to generate a mutant strain (MTL2109) unable to grow on cellobiose and crystalline cellulose, whereas transformation of strain MTL2109 with a CbpA-encoding vector restored growth on both substrates (22, 26).

10.1128/mBio.02206-21.4 FIG S2 Nonlinear (Michaelis-Menten) regression analysis of the activities of the selected metabolic enzymes. (a) Analysis of the phosphorylating activity of the hexokinase on glucose in presence of 25 mM ATP (black squares and line) or 2 mM GTP (red circles and line). (b) Analysis of the hydrolytic activity of the hexokinase on ATP in presence of 5 mM glucose. (c) Analysis of the hydrolytic activity of the hexokinase on GTP in presence of 5 mM glucose. (d) Analysis of the phosphorylating activity of the hexokinase on mannose in presence of 25 mM ATP (black squares and line or 2 mM GTP (red circles and line). (e) Analysis of the activity of cellobiose phosphorylase on cellobiose in presence of 0, 1, or 5 mM glucose. (f) Analysis of the phosphorylating activity of the galactokinase on galactose in presence of 25 mM ATP (black squares and line) or 5 mM GTP (red circles and line). (g) Analysis of the phosphorylating activity of the galactokinase in presence of 100 mM galactose and variable concentrations of ATP. (h) Analysis of the phosphorylating activity of the galactokinase in presence of 100 mM galactose and variable concentrations of GTP. (i) Analysis of the activity of the xylose isomerase on xylose. (j) analysis of the activity of the xylose isomerase on xylulose. (k) Analysis of the activity of the α-phosphoglucomutase using Glu1P as the substrate. (l) Analysis of the activity of the α-phosphoglucomutase using Glu6P as the substrate. The activities were monitored by HPAEC-PAD, using a PA1 column (a [ATP]; d [ATP]; and e, k, and l) or using a refractive index detector and an Aminex HPX87H column (a [GTP]; b, c, and d [GTP]; f, g, h, i, and j). The data show the means of two to four independent experiments, and bars indicate the standard deviations. Curves fitting was performed using the Origin 2019b software. Download FIG S2, DOCX file, 0.1 MB. Copyright © 2021 Kampik et al. 2021 Kampik et al. https://creativecommons.org/licenses/by/4.0/ This content is distributed under the terms of the Creative Commons Attribution 4.0 International license.

The hexokinase which phosphorylates glucose to Glu6P displays a low *K_m_* for glucose (Table 1; see also Fig. S2a) and an elevated *k*_cat_/*K_m_* value in the presence of 25 mM ATP. Nevertheless, the estimated *K_m_* for ATP at 32.2 mM was unexpectedly high (Table 1; see also Fig. S2b). This value is well above the intracellular concentration of ATP, which we extrapolated at 1.5 to 2 mM on the basis of former studies (27, 28), and much higher than those reported for other ATP-dependent bacterial glucose kinases and hexokinases (ranging from 0.05 to 1.1 mM) (19). This unexpected result prompted us to assay its activity in the presence of GTP. Clearly, the hexokinase exhibits a strong preference for GTP as the *K_m_* value for GTP (Table 1; see also Fig. S2c) is 80-fold lower than that estimated for ATP. Determination of the catalytic parameters for glucose at saturating concentration of GTP led to a *k*_cat_/*K_m_* value of 42,000 IU μmol^−1^ mM^−1^ (Table 1; see also Fig. S2a). To date, GTP-dependent glucose kinases were described for only two thermophiles (29, 30). Our data demonstrate that a mesophilic bacterium can also synthesize a GTP-dependent hexokinase. The *R. cellulolyticum* hexokinase was further found to phosphorylate mannose with catalytic parameters similar to those established for glucose (Table 1; see also Fig. S2d). Its activity was not altered by the presence of xylose, galactose or cellobiose at high concentrations.

**FIG 2:**
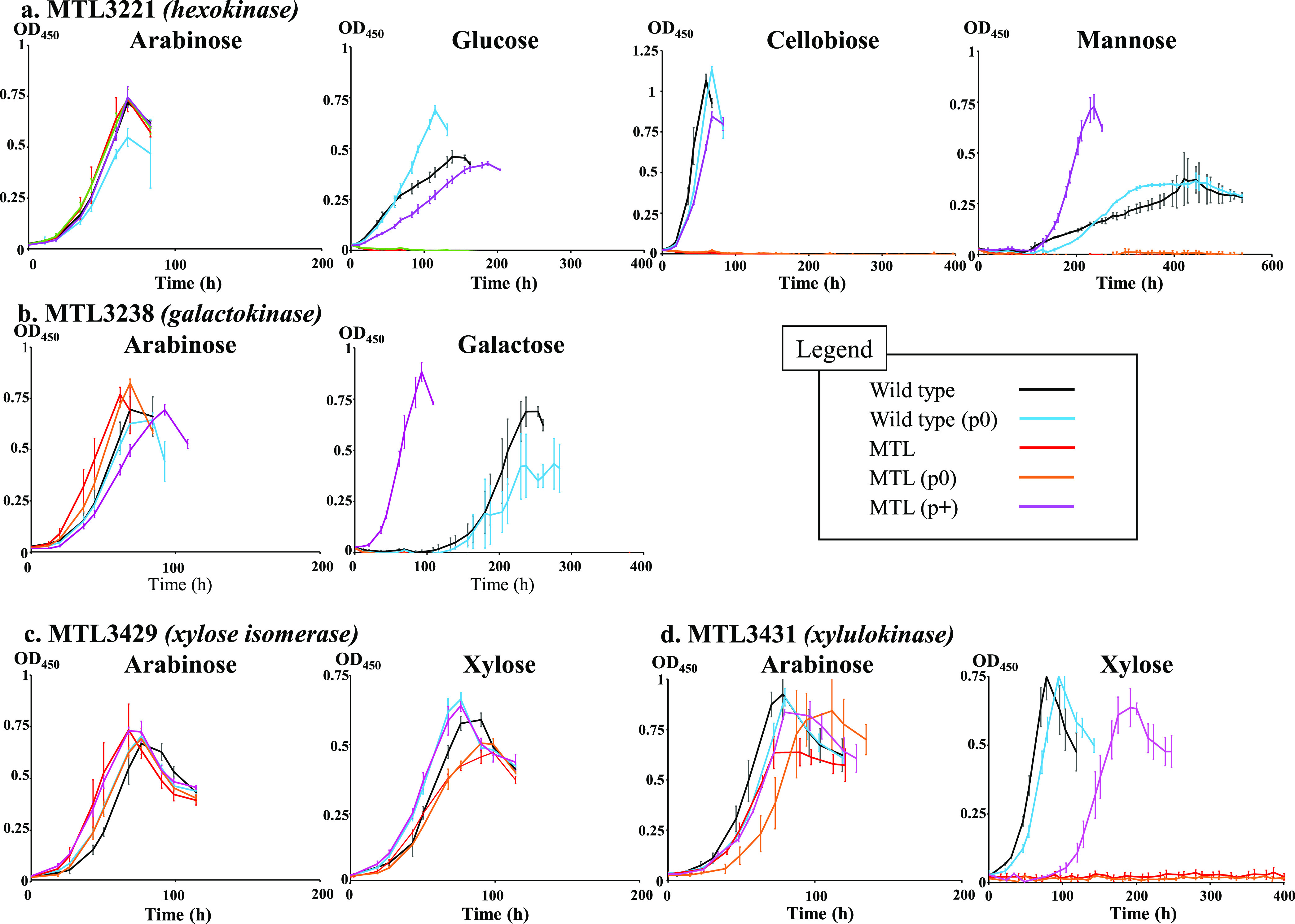
Growth of the various mutant and wild-type strains on simple sugars (2 g/liter). (a) MTL3221 (hexokinase-encoding gene inactivated) and wild-type strains on arabinose-, glucose-, cellobiose-, and mannose-based media. (b) MTL3238 (galactokinase-encoding gene inactivated) and wild-type strains on arabinose- and galactose-based medium. (c) MTL3429 (xylose isomerase-encoding gene inactivated) and wild-type strains on arabinose- and xylose-based media. (d) MTL3431 (xylulokinase-encoding gene inactivated) and wild-type strains on arabinose- and xylose-based media. All cultures were inoculated (1/20) with precultures grown on arabinose-based medium. In all cases, wild-type strain (black), wild-type strain carrying the empty vector p0 (blue), mutant strain (red), mutant strain carrying the empty vector p0 (orange), and mutant strain harboring the complementation vector (purple) are shown. The carbon source is indicated at the top of each graph. In the graphical legend, p0 and *P*+ designate the empty expression vector and the vector used for complementation, respectively. The data show the means of three biological replicates, and bars indicate the standard deviations.

Inactivation of its encoding gene at locus Ccel_3221 was successfully conducted using the ClosTron technology (25) (see Fig. S3). The resulting mutant strains, MTL3221 and MTL3221(p0) carrying an empty expression vector, were expectedly unable to grow on glucose- and mannose-based medium, but also on cellobiose-based medium (Fig. 2a), thereby indicating that the hexokinase has an essential role in preventing the feedback inhibition of CbpA by glucose. Transformation of the MTL3221 strain with a vector encoding the hexokinase restored its growth on these substrates.

10.1128/mBio.02206-21.5 FIG S3 Southern blot analyses of the various mutant strains. Genomic DNA of the wild-type and mutant strains and plasmidic DNA of pMTL-3431 were digested with the restriction enzymes indicated above the lanes and subjected to gel electrophoresis and transfer, as previously described (18). The blot was then subjected to hybridization using a labeled probe targeting the erythromycin cassette present in the intron. (A) Analysis of mutant strains MTL3238 and MTL3221. The presence of single bands at the expected sizes 3.7 bp (EcoRI), 6.7 bp (EcoRV), and 3.0 bp (HindIII) for MTL3238 and at 4.6 bp (EcoRI), 6.6 bp (EcoRV), and 2.8 bp (HindIII) for MTL3221 demonstrated single intron integrations in the respective target genes. (B) Analysis of mutant strain MTL3429. The presence of single bands at the expected sizes 3.6 bp (EcoRV) and 4.4 bp (HindIII) demonstrated a single intron integration in the target gene. The size of the observed band for the restriction enzyme HaeIII indicated that the downstream HaeIII site closest to the intron in this strain was not recognized, leading to a DNA fragment of 4.1 bp (between the closest upstream HaeIII site and the next downstream HaeIII site) instead of 2.9 bp. (C) Analysis of mutant strain MTL3431. The size of the observed bands indicated that the gene at locus Ccel_3431 had been interrupted by the single integration of plasmid pMTL-3431 used for the strain construction within the target gene, as illustrated in panel D. (D) Schematic representation of genetic regions of interest in relation to the construction of the xylulokinase knockout mutant MTL3431. Genes are shown in black, introns of the Clostron technology in white, and the backbone of the plasmid pMTL-3431 in gray. The latter is not drawn proportional in size, as indicated by dashed gray lines. Restriction sites relevant for DNA fragments after hybridization with the probe are indicated for pMTL-3431 (a), the result of the intron insertion without recombination (b), and the result of the vector integration after a recombination event, which can lead to two schemes (c and d). The DNA fragments obtained after hybridization are represented as dashed lines, and their size is given in kilobases in brackets below the lines. The results indicated that the xylulokinase mutant strain corresponds to the genotype with a plasmid integration as shown in panel d. Download FIG S3, DOCX file, 0.1 MB. Copyright © 2021 Kampik et al. 2021 Kampik et al. https://creativecommons.org/licenses/by/4.0/ This content is distributed under the terms of the Creative Commons Attribution 4.0 International license.

The galactokinase, which phosphorylates α-galactose to α-galactose 1-P (Gal1P), also displays unconventional enzymatic parameters (Table 1; see also Fig. S2f and g). The enzyme exhibits a high catalytic velocity but has estimated *K_m_* values toward galactose and ATP of 20.55 and 13.71 mM, respectively, far exceeding the *K_m_* values reported to date for bacterial galactokinases (31, 32). Furthermore, the galactokinase also displays a clear preference for GTP over ATP, reflected in its 13-fold lower *K_m_* for GTP compared to that for ATP (Table 1; see also Fig. S2g and h). At a saturating concentration of GTP, the *K_m_* for galactose remains similar to that determined in the presence of ATP, whereas the *k*_cat_ is 2-fold reduced (Table 1). Only one GTP-dependent galactokinase was to date reported for the thermophile (*Pseudo*)*clostridium thermosuccinogenes* (33), with whom the *R. cellulolyticum* kinase shares 62% sequence identity. The galactokinase activity was not impacted in the presence of glucose, cellobiose, or xylose at concentrations up to 20 mM.

Inactivation of the gene encoding the galactokinase (see Fig. S3) was performed as described above. The resulting mutant strains MTL3238 and MTL3238(p0) were no longer able to grow on galactose-based medium (Fig. 2b). The gene encoding the galactokinase likely forms an operon with the downstream gene at locus Ccel_3237 predicted to encode another enzyme of the Leloir pathway, the UDP-galactose 4-epimerase (34) (Fig. 1b). Thus, in strains MTL3238 and MTL3238(p0), due to a probable polar effect (35, 36), the expression of this second gene may be considerably diminished. Complementation of strain MTL3238 therefore implied the transformation with a vector harboring both genes. The resulting strain MTL3238(p3238-3237) was found to grow even faster than the control strains on galactose (Fig. 2b), presumably because of the plasmid-based overexpression of these genes.

The fourth selected enzyme, the xylose isomerase encoded by the gene at locus Ccel_3429, catalyzes the reversible conversion of xylose to xylulose and requires MgCl_2_, MnCl_2_, or CoCl_2_ to be fully active. The determination of the kinetic parameters (in the presence of MgCl_2_) revealed significantly higher *K_m_* values (Table 1; see also Fig. S2i and j) for both xylose and xylulose compared to most other known bacterial xylose isomerases (37). The selected enzyme catalyzes the conversion of xylulose to xylose with a 7-fold higher “catalytic efficiency” (*k*_cat_/*K_m_*) than the reverse isomerization. The activity of the enzyme was neither inhibited nor enhanced in the presence of 20 mM glucose, cellobiose, or galactose.

Inactivation of its encoding gene was performed. However, the resulting mutant strains, MTL3429 and MTL3429(p0), were both found to display an altered but significant growth on xylose-based medium (Fig. 2c), thereby indicating that (an)other enzyme(s) in *R. cellulolyticum* exhibit(s) xylose isomerase activity. Nevertheless, transformation of strain MTL3429 with a vector carrying the xylose isomerase-encoding gene restored a wild-type-like growth in xylose-based medium (Fig. 2c).

This observation prompted us to inactivate the sole gene predicted to encode the xylulokinase (38) at locus Ccel_3431 (Fig. 1b), and the resulting mutant strains MTL3431 and MTL3431(p0) were both found unable to utilize xylose (Fig. 2d). As mentioned above, this gene is the first gene of a putative three-gene operon, encoding also a Repressor Orf Kinase (ROK) and the above-mentioned xylose isomerase (Fig. 1b). Integration of the type II intron in the xylulokinase-encoding gene was therefore expected to strongly impact the expression of the two downstream genes. Transformation of strain MTL3431 was thus performed with a vector hosting the three-gene operon (p3431-3430-3429) and restored growth on xylose (Fig. 2d).

The α-phosphoglucomutase, which catalyzes the reversible conversion of Glu1P to Glu6P (24), exhibits more favorable kinetic parameters for the isomerization of Glu1P (Table 1). The determined *K_m_* and *k*_cat_ values (Table 1; see also Fig. S2k and l) are in the range of those reported for other bacterial α-phosphoglucomutases (39–41).

Inactivation of its encoding gene using the ClosTron technology could not be achieved despite the use of three different integration sites within the targeted gene sequence. This observation suggests that this gene may be essential or that the local DNA topology is not suitable for type II intron integration.

The *k*_cat_/*K_m_* ratios of the various selected enzymes (Table 1) exhibit very large discrepancies, suggesting that the hexokinase primarily catabolizes glucose generated during the depolymerization of xylogluco-oligosaccharides, thereby reducing the amount of glucose to a subinhibitory concentration with respect to CbpA. The latter would subsequently degrade the increasing concentration of cellobiose to glucose and Glu1P, while cytosolic galactose and xylose would continue to accumulate. Finally, their concentration would reach a threshold allowing the galactokinase and the xylose isomerase, which both display elevated *K_m_* values, to process these sugars at a significant velocity. To assess this hypothesis, the five purified enzymes were combined in physiological proportions and assayed on various mixtures of simple sugars.

### Activity on mixes of simple sugars mimicking the imported four-glucosyl xylogluco-oligosaccharides.

A crude extract of wild-type *R. cellulolyticum* was prepared from a culture on xyloglucan at the late exponential phase. The concentration of each selected enzyme in the extract was then estimated on the basis of its activity and is reported in Table 2. Indeed, for the xylose isomerase, the estimated concentration corresponds to the overall concentration of enzymes displaying xylose isomerase activities, since at least one other enzyme can catalyze the isomerization of xylose into xylulose (see above).

**TABLE 2:** Concentrations of targeted metabolic enzymes in *R. cellulolyticum* crude extract

Enzyme	Mean concn (nM) ± the SD	Normalized concn^*a*^
α-Phosphoglucomutase	271 ± 84	1
Hexokinase	544 ± 21	2
Galactokinase	1,186 ± 20	4.5
Cellobiose phosphorylase	6,750 ± 1,054	25
Xylose isomerase	39,830 ± 589	146.5

a The concentrations are normalized based on that of α-phosphoglucomutase.

**FIG 3:**
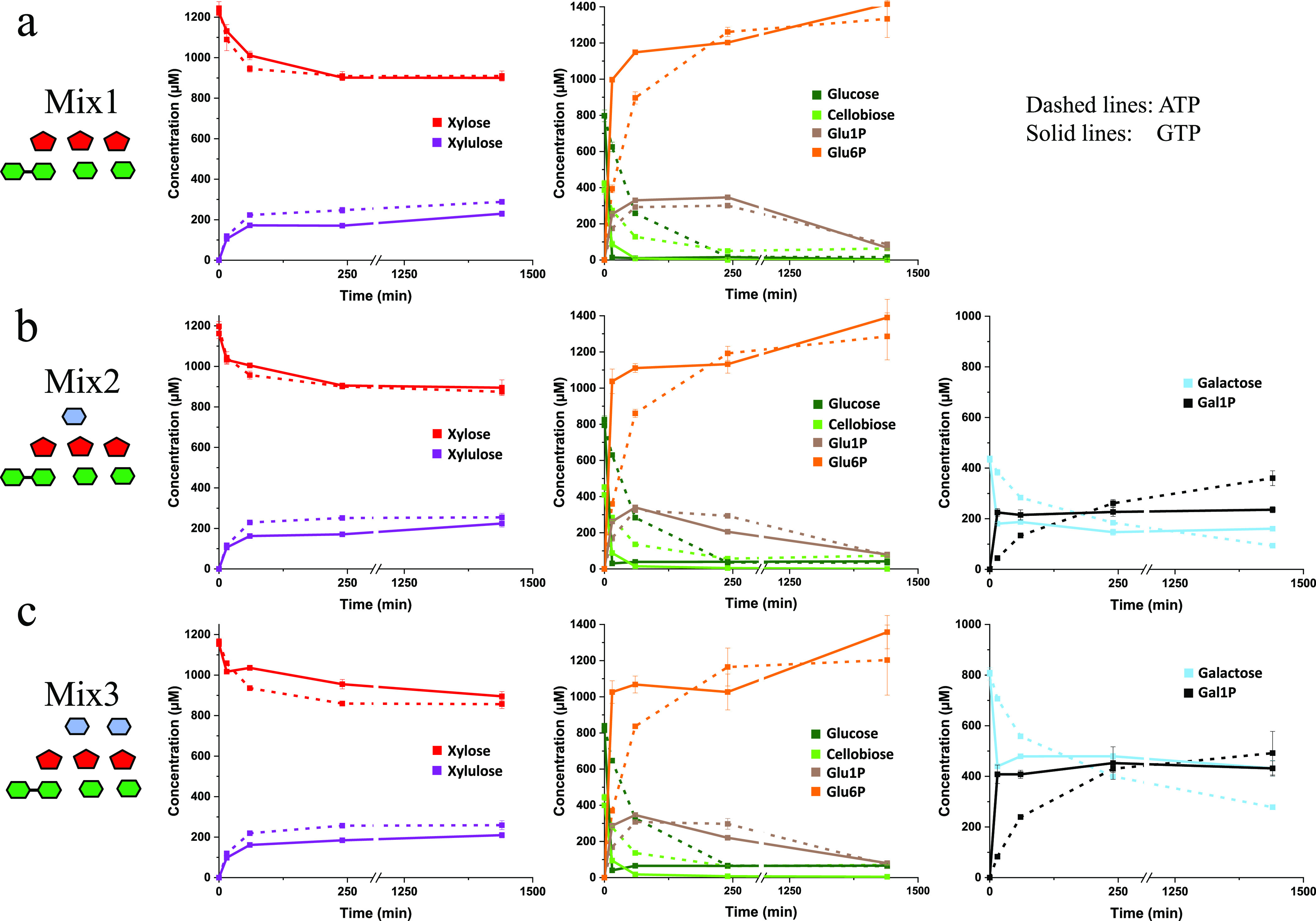
Activity in the presence of 2 mM ATP or GTP at 37°C of the combination of pure metabolic enzymes on mixes of simple sugars mimicking the three types of xylogluco-oligosaccharides imported by *R. cellulolyticum*. (a) Sugar mix 1 containing 0.4 mM cellobiose, 0.8 mM glucose, and 1.2 mM xylose. (b) Sugar mix 2 containing 0.4 mM cellobiose, 0.8 mM glucose, 1.2 mM xylose, and 0.4 mM galactose. (c) Sugar mix 3 containing 0.4 mM cellobiose, 0.8 mM glucose, 1.2 mM xylose, and 0.8 mM galactose. In all cases, the enzymes were at final concentrations of 1 μM for xylose isomerase, 171 nM for cellobiose phosphorylase, 30.5 nM for galactokinase, 13.85 nM for hexokinase, and 6.85 nM for α-phosphoglucomutase. For all experiments, the buffer was 25 mM HEPES (pH 7.0) containing 10 mM KHPO_4_, 5 mM MgCl_2_, and either 2 mM ATP (dashed lines) or 2 mM GTP (solid lines). At each time point, detection and quantification of sugars was performed by HPAEC-PAD using a PA20 column (and a PA1 column for experiments in the presence of GTP) and by HPLC-RI on an Aminex HPX87H coupled with an RI detector. The data show the means of four independent replicates, and bars indicate the standard deviations. Symbols are as defined for Fig. 1.

To assess the aforementioned hypothesis, the five purified enzymes were combined according to the normalized concentrations (Table 2). The enzymatic mixture was assayed at 37°C on three different mixes of simple sugars mimicking the three different types of four-glucosyl-backbone xylogluco-oligosaccharides (XGO_4_) containing 0, 1, or 2 galactosyl residues, known to be imported and depolymerized by *R. cellulolyticum* (9). The “xylogluco-oligosaccharide equivalent” concentration was set at 0.4 mM (i.e., the mixes contained 0.4 mM cellobiose, 0.8 mM glucose, 1.2 mM xylose, and galactose at 0, 0.4, or 0.8 mM). The mixes also contained 2 mM either ATP or GTP (Fig. 3), which is theoretically sufficient for complete phosphorylation of the hexose contents. The consumption of the substrates and the release of the products were monitored for 24 h. In the presence of ATP (Fig. 3, dashed lines), all sugars are consumed simultaneously but at different velocities. Glucose and cellobiose are the most rapidly catabolized but, in contrast to our initial hypothesis, at seemingly similar rates. After 1 h of incubation, 60 to 70% of cellobiose and glucose contents were processed, whereas galactose and xylose were consumed at only 30 to 35% and 20%, respectively. At longer incubation times, larger fractions of galactose were catabolized, whereas the proportion of consumed xylose remained stable at around 20 to 25%. Glu1P generated by CbpA accumulated at the beginning of the kinetics but was completely converted by the α-phosphoglucomutase at longer incubation times. The absence or presence of galactose at up to 0.8 mM had no significant impact on the other enzymatic reactions, which occurred at similar velocities for all mixes.

In the presence of GTP (Fig. 3, solid lines), the consumption of glucose and galactose was unsurprisingly much faster than with ATP. Cellobiose was also processed faster, though its breakdown into glucose and Glu1P by CbpA does not require any NTP. This result probably reflects the fact that in the presence of GTP, the glucose content is very rapidly diminished by the hexokinase to a subinhibitory concentration with respect to CbpA, whereas with ATP, the concentration of glucose remained high enough at the beginning of the kinetics to induce a significant inhibition of CbpA. As observed with ATP, the fraction of xylose isomerized to xylulose remained at 20 to 25% after 24 h. This low consumption for both experimental conditions probably reflects the weak activity of the xylose isomerase. As the selected enzyme more efficiently catalyzes the isomerization of xylulose to xylose (Table 1), in the absence of any xylulose-consuming enzyme, a stable xylose/xylulose equilibrium is reached after 4 h of incubation.

**FIG 4:**
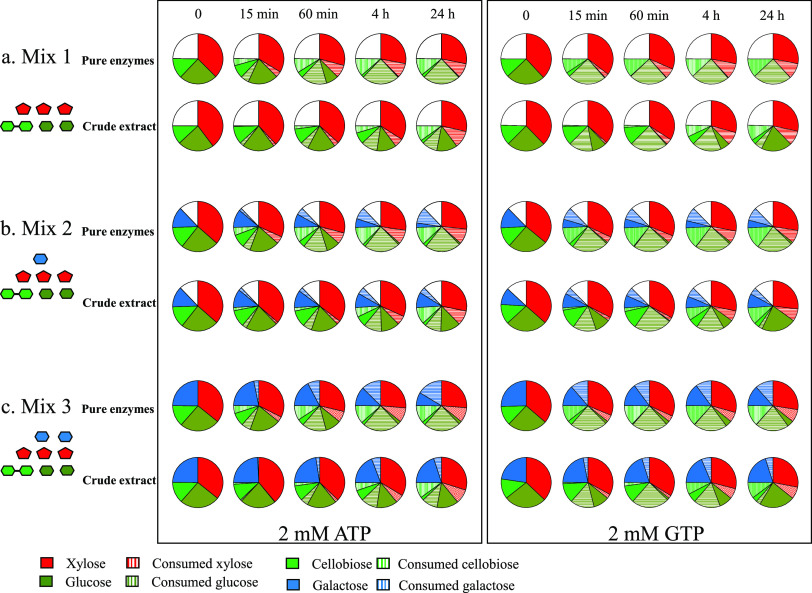
Comparison between crude extract and combination of pure enzymes on mixes of simple sugars mimicking the three types of xylogluco-oligosaccharides imported by *R. cellulolyticum*. (a) Sugar mix 1 containing 0.4 mM cellobiose, 0.8 mM glucose, and 1.2 mM xylose. (b) Sugar mix 2, same as sugar mix 1 but supplemented with 0.4 mM galactose. (c) Sugar mix 3, same as sugar mix 1 but supplemented with 0.8 mM galactose. Experimental conditions are the same as for Fig. 3, except that for the crude extract 12.5 μl was mixed with 478.5 μl of sugar mix. Incubation times are shown at the top of each column, and the type of NTP at 2 mM is indicated at the bottom. For the crude extract, the detection and quantification of sugars were performed by HPAEC-PAD using a PA20 column. For the combination of pure enzymes, the data are from Fig. 3. The data show the means for the crude extracts of three or four independent replicates (standard deviations were within 10%). Symbols are as defined for Fig. 1.

Similar experiments were conducted using the wild-type crude extract instead of the five-enzyme combination. The volume of injected extract in the reaction tubes was adjusted to provide the selected metabolic enzymes at concentrations similar to those of the five-enzyme combination. The data (Fig. 4) show that all sugars are again catabolized collectively but at different velocities by the crude extract. However, all enzymatic reactions were slower for the crude extract, in particular the phosphorylation of the hexoses, whose consumed fractions were markedly lower for all incubation times. This difference is probably attributable to the presence of multiple other NTP-hydrolyzing enzymes in the extract, thereby reducing the amount of available NTP for the kinases. Unsurprisingly, the consumption of glucose and galactose by the crude extract was faster in the presence of GTP compared to ATP, and the conversion of cellobiose was also slightly improved. Nevertheless, while glucose was nearly totally consumed by the crude extract after 1 h of incubation with GTP, the concentration of glucose unexpectedly rose again after 4 h of incubation, and the initial pool of glucose (0.8 mM) was almost completely reestablished at 24 h. This surprising surge in glucose with the crude extract was not detected in the presence of ATP. Moreover, the five-enzyme combination also failed to generate glucose at long incubation times with both GTP and ATP.

**FIG 5:**
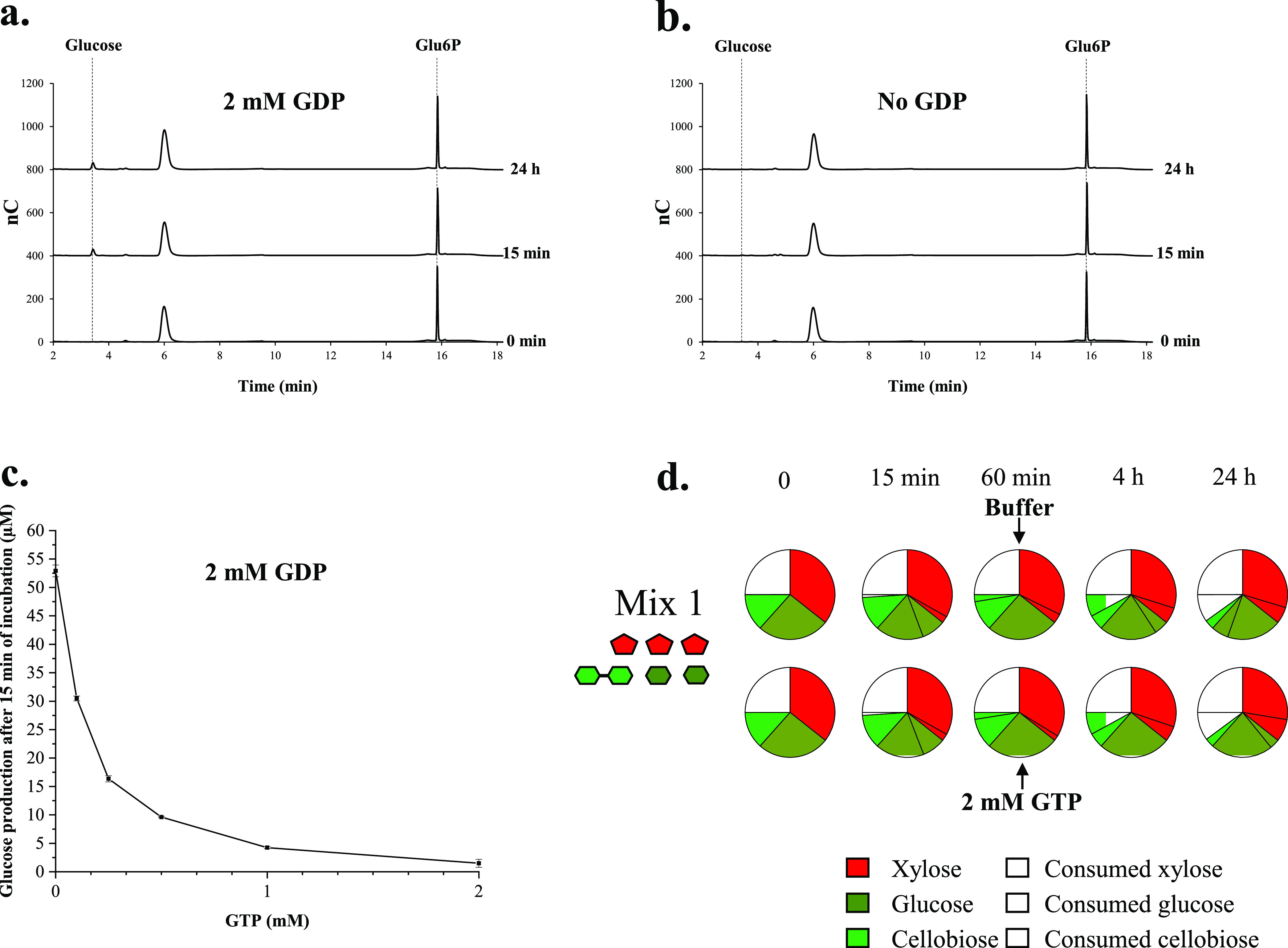
Hexokinase activity on Glu6P and impact of GTP. (a) The activity of the hexokinase (final concentration, 13.85 nM) was monitored at 37°C on 1 mM Glu6P in the presence of 2 mM GDP in 25 mM HEPES (pH 7.0) buffer containing 10 mM KHPO_4_ and 5 mM MgCl_2_. Samples pipetted at various incubation times (0 min, 15 min, 60 min, 4 h, and 24 h) were analyzed by HPAEC-PAD using a PA1 column, but only the chromatograms obtained at 0 min, 15 min, and 24 h are shown (intermediate time points displayed highly similar patterns and glucose concentration). The retention times of glucose and Glu6P are shown (dotted line), and the incubation times are indicated on the right side of each chromatogram. (b) Same as panel a except that GDP was omitted. (c) The activity of the hexokinase (final concentration, 13.85 nM) was monitored at 37°C for 15 min on 1 mM Glu6P in the presence of 2 mM GDP (same buffer as for panel a) and various concentrations of GTP ranging from 0 to 2 mM. At the end of the incubation, the glucose concentration was determined by HPAEC-PAD using a PA1 column. The graph shows the amount of detected glucose as a function of the GTP concentration. The data show the means of two independent replicates, and bars represent the standard deviations. (d) Activity of the crude extract on sugar mix 1 (containing 0.4 mM cellobiose, 0.8 mM glucose, and 1.2 mM xylose) was performed using the same experimental conditions as in Fig. 4a except that either 20 μl of 20 mM HEPES (pH 7.0) buffer or 20 μl of 50 mM GTP buffered at pH 7.0 (final concentration, 2 mM) was injected after 60 min of incubation (indicated by an arrow). Detection and quantification of sugars was performed by HPAEC-PAD using a PA1 column. Incubation times are indicated at the top of each column. The data show the means of three independent replicates (standard deviations were within 10%). Symbols are as defined for Fig. 1.

This result prompted us to investigate whether the hexokinase might catalyze the reverse reaction, though this category of enzyme is generally considered “virtually” irreversible (42). This hypothesis was confirmed by the data reported in Fig. 5a in the presence of 2 mM GDP; the hexokinase produced 55 μM glucose from 1 mM Glu6P within 15 min, whereas no production was observed without GDP (Fig. 5b). The absence of a detectable reverse reaction with the five-enzyme combination could perhaps be attributable to the presence of significant amounts of available GTP, even after 24 h of incubation (estimated to be ≥0.4 mM), which may prevent this activity. In contrast, to the crude extract, depletion of the GTP pool is presumably more complete and faster because of the presence of multiple NTP-consuming enzymes. Thus, the dephosphorylation activity of the hexokinase on Glu6P was reexamined with 2 mM GDP and variable concentrations of GTP (Fig. 5c) and showed that GTP prevents the reverse reaction from occurring, even at a low concentration. A complementary experiment was conducted using the crude extract and sugar mix 1 containing 2 mM GTP. In this experiment, a second pulse of 2 mM GTP was performed after 1 h of incubation (Fig. 5d), when nearly all glucose is consumed and before the reverse reaction is observed. Under these conditions, the resurgence of glucose was no longer detected after 4 h and reduced by 83% after 24 h compared to the control experiment in which only buffer was injected. Altogether, these data support the hypothesis that the fast depletion of the GTP pool occurring with the crude extract triggers the dephosphorylation of Glu6P and consequently the production of GTP by the hexokinase.

### Growth of wild-type and mutant strains on xyloglucan.

**FIG 6:**
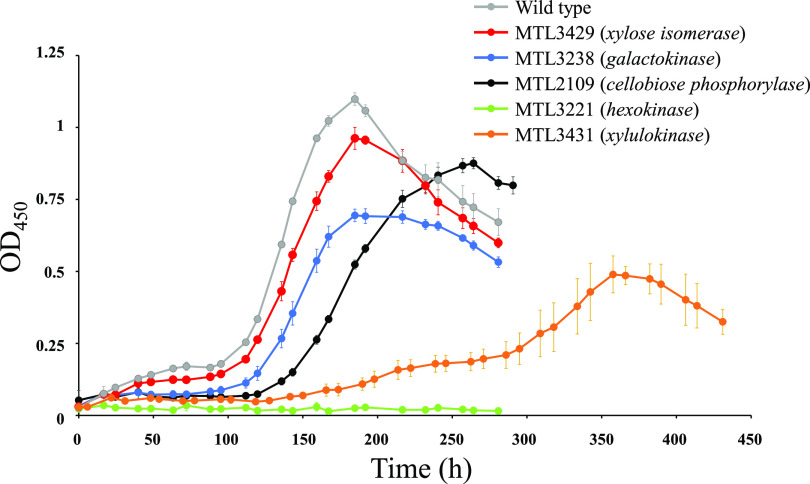
Growth of the wild type and various mutant strains on xyloglucan (3.5 g/liter). The cultures were inoculated (1/20) with arabinose (2 g/liter)-grown precultures. The growths of wild-type (gray), MTL3221 (green, hexokinase-encoding gene inactivated), MTL3238 (blue, galactokinase-encoding gene inactivated), MTL2109 (black, cellobiose phosphorylase-encoding gene inactivated), MTL3429 (red, xylose isomerase-encoding gene inactivated), and MTL3431 (orange, xylulokinase-encoding gene inactivated) are shown. The data show the means of four independent experiments. Bars represent the standard deviations.

The growth of the various strains in xyloglucan-based medium was examined (Fig. 6).

Surprisingly, no growth was detected for strain MTL3221, which lacks the hexokinase. This enzyme was shown to be essential for the utilization of glucose and indirectly cellobiose by preventing feedback inhibition of CbpA by glucose. The absence of this pivotal enzyme was therefore projected to induce a strong impact on xyloglucan utilization. Nevertheless, the mutant strain was also expected to utilize the galactose and xylose released during the xylogluco-oligosaccharides depolymerization and was thus anticipated to display some growth on xyloglucan, which was not observed.

Mutant strain MTL3238 lacking the galactokinase can still utilize xyloglucan (Fig. 6). At the beginning of the growth, this mutant strain displayed a doubling time (19.2 ± 1.19 h) similar to that of the wild type (19.6 ± 0.17 h), but the growth prematurely slowed down and stopped, thereby inducing a 37% decrease in final biomass, although this strain should be able to catabolize 87% of the monosaccharides contained in xyloglucan. Analysis of the culture supernatants of wild-type and MTL3238 strains at midexponential and late exponential phases of growth (see Fig. S4) revealed the presence of galactose, only for the mutant strain. Its concentration evolved from 500 μM at the midexponential phase of growth to 2.4 mM at the end of growth, representing approximately 85% of the estimated total content of galactose in 3.5 g liter^−1^ xyloglucan. These observations may be explained as follows: the solute binding protein of the ABC-transporter importing the xylogluco-oligosaccharides exhibits a 10-fold higher affinity for xylogluco-oligosaccharides displaying no galactosyl decoration (9). Thus, at an early stage of growth, this oligosaccharide is presumably preferentially imported and catabolized by *R. cellulolyticum*, and the lack of galactokinase in strain MTL3238 does not significantly impair the growth compared to the wild-type strain. However, at a later stage of growth, this oligosaccharide becomes depleted, and only xylogluco-oligosaccharides harboring one or two galactosyl decorations are available and imported. The accumulating galactose would then be continuously excreted by strain MTL3238, possibly through an energy-consuming export system, inducing an early stationary phase of growth.

10.1128/mBio.02206-21.6 FIG S4 Analyses of the culture supernatants of wild-type (a) and MTL3238 (b) strains grown on xyloglucan-based medium. Culture supernatants were analyzed by HPAEC-PAD on a PA1 column. Lower curves correspond to mid-exponential phase of growth, and upper curves correspond to late exponential phase of growth. The retention times of galactose and four-glucosyl-backbone xyloglucandextrins (XGO4) are shown (dotted lines). Download FIG S4, DOCX file, 0.04 MB. Copyright © 2021 Kampik et al. 2021 Kampik et al. https://creativecommons.org/licenses/by/4.0/ This content is distributed under the terms of the Creative Commons Attribution 4.0 International license.

The growth of the mutant strain MTL2109 which lacks CbpA was slightly altered compared to the wild-type strain, as reflected by a 1.22-fold increased doubling time and a final biomass reduced by 20%. Clearly, the other sugars released during the depolymerization of xylogluco-oligosaccharides can to a large extent sustain the growth of this mutant strain. No cellobiose was detected in the supernatant of strain MTL2109, suggesting the bacterium lacks a functional cellobiose-export system. Nevertheless, it is worth noting that the β-glucosidase Glu3A (Fig. 1) involved in the cytosolic depolymerization of xylogluco-oligosaccharides was formerly shown to display a weak activity on cellobiose (9), characterized by a *K_m_* exceeding 40 mM. In this mutant strain, Glu3A probably hydrolyses the cellobiose accumulating in the cytosol into two glucoses, although with less efficacy than CbpA. The lower growth rate and yield may also stem from the fact that this alternative pathway involving Glu3A is energetically less favorable: two GTP molecules are required to convert cellobiose into two Glu6P, whereas in the case of the “CbpA pathway” only one GTP is needed to generate two Glu6P.

Unsurprisingly, the MTL3429 mutant strain lacking the selected xylose isomerase displays a barely affected growth on xyloglucan compared to the wild-type strain (Fig. 6), thus confirming that at least one additional enzyme can satisfactorily substitute the absence of the targeted isomerase for the utilization of xyloglucan. In contrast, the disruption of the xylose utilization pathway engendered by the inactivation of the xylulokinase-encoding gene in mutant strain MTL3431 induced to a 2.8-fold increased doubling time and a 55% reduction of final biomass. Such a strong impact on the growth is surprising since this mutant strain should have metabolized the other sugars released during the xylogluco-oligosaccharides depolymerization and was thus anticipated to display a more robust growth. Furthermore, xylose was not found to inhibit any of the metabolic enzymes selected in the present study, but the effect of xylulose was not investigated. Analysis of the culture supernatant of strain MTL3431 (see Fig. S5) indicated that only a small amount of xylose (640 μM) was excreted in the environment, together with 470 μM galactose and 390 μM glucose at the end of the growth, which may only reflect some cell lysis.

10.1128/mBio.02206-21.7 FIG S5 Analyses of the culture supernatants of wild-type (a) and MTL3431 (b) strains grown on xyloglucan-based medium. Culture supernatants were analyzed by HPAEC-PAD on a PA20 column. Lower curves correspond to mid-exponential phase of growth, and upper curves correspond to late exponential phase of growth. The retention times of galactose, glucose, and xylose are shown (dotted lines). Download FIG S5, DOCX file, 0.2 MB. Copyright © 2021 Kampik et al. 2021 Kampik et al. https://creativecommons.org/licenses/by/4.0/ This content is distributed under the terms of the Creative Commons Attribution 4.0 International license.

Altogether, the phenotypes of the various mutant strains on xyloglucan confirmed the hierarchy suggested by the characterization of the selected metabolic enzymes, in particular the prominent role of the hexokinase. Nevertheless, the lack of growth of mutant strain MTL3221 and the strongly impaired growth of mutant strain MTL3431 remained intriguing and prompted us to reexamine the three cytosolic enzymes that depolymerize the xylogluco-oligosaccharides.

### Inhibition of xylogluco-oligosaccharide depolymerization by simple sugars.

The β-galactosidase Gal42A, the β-glucosidase Glu3A, and the α-xylosidase Xyl31A (Fig. 1) were purified as formerly reported (9).

The activity of Gal42A on *p*-nitrophenyl-β-d-galactoside (*p*NPβGal) was not inhibited by xylose or glucose at a concentration of 20 mM. However, galactose is a potent competitive inhibitor, characterized by a *K_i_* value of 2.35 mM (Table 3; see also Fig. S6a), thus indicating that Gal42A is responsive to feedback inhibition. More surprisingly, cellobiose, which is neither a substrate nor a product of Gal32A, acts as a noncompetitive inhibitor, with a *K_i_* value of 19.28 mM.

**TABLE 3:** Inhibitors of the three cytoplasmic enzymes in charge of the depolymerization of the imported four-glucosyl-backbone xylogluco-oligosaccharides (XGO_4_)

Sugar	Mean enzyme concn (mM) ± SD^*a*^		
	β-Galactosidase Gal42A	β-Glucosidase (Glu3A)	α-Xylosidase (Xyl31A)
Galactose	2.35 ± 0.05 (C)	NI	NI
Cellobiose	19.28 ± 1.41 (NC)	36.34 ± 5.57 (C)	NI
Glucose	NI	3.99 ± 0.33 (C)	1.83 ± 0.42 (C)
Xylose	NI	NI	NI

a The type of inhibition (see Fig. S4) is indicated: C, competitive; NC, noncompetitive; NI, no inhibition observed.

10.1128/mBio.02206-21.8 FIG S6 Inhibitions of the three cytoplasmic xylogluco-oligosaccharide-depolymerizing enzymes. (a) Inhibition of the β-galactosidase Gal42A by galactose and cellobiose. Activity was monitored spectrophotometrically (*A*_400_) on the chromogenic substrate *p*-nitrophenyl-β-d-galactoside (*p*NPβGal). (b) Inhibition of the β-glucosidase Glu3A by glucose and cellobiose. Activity was monitored spectrophotometrically (*A*_400_) on the chromogenic substrate *p*-nitrophenyl-β-d-glucoside (*p*NPβGlu). (c) Inhibition of the α-xylosidase Xyl31A by glucose. Activity was monitored on the disaccharide isoprimeverose by HPAEC-PAD using a PA1 column. The data show the means of two independent experiments, and bars represent the standard deviations. Curves fitting was performed using Origin 2019b software. Download FIG S6, DOCX file, 0.10 MB. Copyright © 2021 Kampik et al. 2021 Kampik et al. https://creativecommons.org/licenses/by/4.0/ This content is distributed under the terms of the Creative Commons Attribution 4.0 International license.

The hydrolytic activity of Glu3A on *p*-nitrophenyl-β-d-glucoside (*p*NPβGlu) was not altered in the presence of 20 mM xylose or galactose. However, glucose and cellobiose were both found to act as feedback competitive inhibitors of Glu3A. The *K_i_* value for cellobiose, however, is 9-fold higher than that for glucose (Table 3; see also Fig. S6b).

Xylose, cellobiose, and galactose at 20 mM were not found to inhibit Xyl31A when assayed on the disaccharide 6-*O*-(α-d-xylopyranosyl)-d-glucose (isoprimeverose), whereas glucose is a competitive inhibitor of Xyl31A, with a *K_i_* value of 1.83 mM (Table 3; see also Fig. S6c).

Thus, the activity of the three enzymes that depolymerize the imported xylogluco-oligosaccharides is tightly controlled by the cytosolic concentrations of galactose, cellobiose, and glucose through a network of competitive and noncompetitive inhibitions. These observations also shed new lights on the unexpected absence of growth of strain MTL3221 on xyloglucan, which would be due to a cascade of inhibitions: the lack of a functional hexokinase in this strain would induce an accumulation of cytosolic glucose, which would inhibit CbpA and consequently trigger an accumulation of cellobiose. The high concentrations of glucose and cellobiose would subsequently act in concert to inhibit Gal32A, Glu3A, and Xyl31A, thereby inducing a shutdown of the xylogluco-oligosaccharide depolymerization process and preventing strain MTL3221 from utilizing xyloglucan.

These results also suggest that the massive excretion of galactose observed for the strain MTL3238 lacking the galactokinase efficiently prevents the shutdown of the depolymerization of xylogluco-oligosaccharides hosting galactosyl decorations. The resulting diminution of the galactose cytosolic content to subinhibitory levels would thus allow Gal42A to hydrolyze the galactosyl decorations, a prerequisite for subsequent depolymerization of the xylogluco-oligosaccharides by Xyl31A and Glu3A (9). Finally, xylose was not found to inhibit any of the enzymes in charge of this process.

## DISCUSSION

The aim of the present study was to decipher how a bacterium manages the release of multiple metabolizable sugars in the cytoplasmic compartment. For this purpose, we investigated the utilization of a highly decorated polysaccharide, xyloglucan, by *R. cellulolyticum*, which was formerly shown to imply the release of glucose, cellobiose, galactose, and xylose in the cytosol. We applied a combination of biochemical and genetic approaches to target the enzymes catalyzing the first steps of each required metabolic pathway. Quite surprisingly, the hexokinase of the glycolytic pathway and the galactokinase of the Leloir pathway were both found to display a very clear preference for GTP over ATP, though similar GTP-dependent enzymes have only been reported for two thermophilic bacteria to date (29, 30, 33).

The characterization of the targeted metabolic enzymes, whose catalytic efficiencies vary tremendously, suggested a hierarchy among the examined pathways, the glycolytic pathway seeming the most efficient. Nevertheless, our data using a mix of the selected purified enzymes or a crude extract indicate that, although glucose is prioritized, *R. cellulolyticum* consumes the four released sugars collectively but at different velocities. Glucose and cellobiose were found to be the most rapidly processed, followed by galactose and finally xylose. The targeted hexokinase of the glycolytic pathway is the most active and critical enzyme, since inactivation of its gene leads to a mutant strain unable to grow on and catabolize xyloglucan. In contrast, all other mutant strains displayed growth on xyloglucan, although altered to various extents.

**FIG 7:**
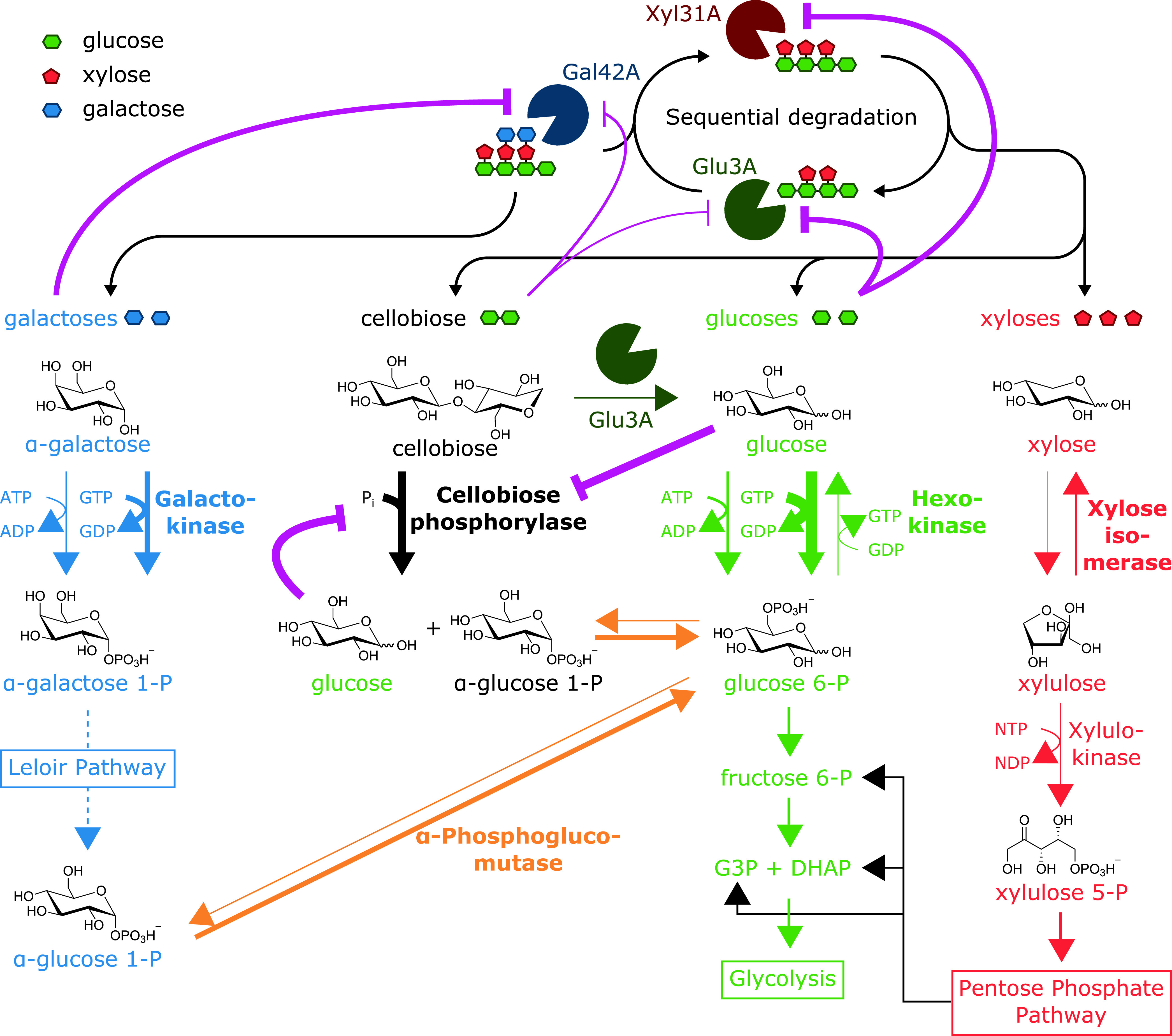
Model of xyloglucan catabolism in *R. cellulolyticum*. Symbols are as defined for Fig. 1. The depolymerization of xyloglucan oligosaccharides and the required downstream metabolic pathways for galactose (blue), cellobiose (black), glucose (green), and xylose (red) catabolism are shown. The enzyme in charge of the reversible conversion of α-glucose 1-P into glucose 6-P is depicted in orange. The names of the characterized enzymes are shown in boldface. The thickness of the arrows depicting the enzymatic reactions reflects the velocity of the reaction. The thickness of the pink lines symbolizing inhibitions is inversely proportional to the determined *K_i_*.

The intriguing absence of growth of the mutant strain lacking the hexokinase prompted us to reexamine the cytosolic depolymerization of the xylogluco-oligosaccharides. We observed that the activity of the three depolymerizing enzymes is controlled by the cytosolic concentrations of galactose, cellobiose, and glucose through an intricate network of competitive and noncompetitive inhibitions (Fig. 7), strongly suggesting that the inability of the mutant strain lacking the hexokinase to grow on xyloglucan is due to a cascade of inhibitions. In contrast, xylose was not found to inhibit (or stimulate) any of the involved enzymes. Thus, in strain MTL3431 lacking the xylulokinase and exhibiting severely impaired growth rate and yield, the depolymerization of the xylogluco-oligosaccharides is likely to function at a normal speed, providing glucose, galactose, and cellobiose that should have sustained a more robust growth. One possible hypothesis is that in this particular context, a very massive cytosolic accumulation of xylose and/or xylulose occurs that would finally impede various biological processes.

Two particularly striking aspects of the overall strategy developed by *R. cellulolyticum* to utilize xyloglucan are the energy-saving and the effective prevention of any carbon overflow. The import of large xylogluco-oligosaccharides through a highly specific ABC-transporter allows their uptake at low concentration in the environment but also at a low ATP cost, considering that these oligosaccharides can contain up to nine metabolizable monosaccharides. Furthermore, cellobiose—and not exclusively glucose—is generated during the depolymerization of the xylogluco-oligosaccharides by the cytoplasmic β-glucosidase (9). The disaccharide is subsequently mainly processed by CbpA (and not by the β-glucosidase Glu3A), which generates Glu1P allowing the saving of another NTP molecule when the phosphorylated hexose enters the glycolytic pathway after isomerization into Glu6P.

The prevention of carbon overflow mainly relies on the slowdown/shutdown of the xylogluco-oligosaccharide cytosolic depolymerization (Fig. 7) when galactose and/or cellobiose and/or glucose accumulate in the cytosol. Another regulation occurs at a downstream level, since glucose inhibits CbpA at low cytosolic concentrations (Fig. 7). The GTP pool is also expected to be a key player. The availability of GTP not only dictates the rate of consumption of galactose and glucose, but, as mentioned above, can also induce the reversion of the first step of glycolysis. The resulting cytosolic production of glucose would in turn inhibit the consumption of cellobiose and the depolymerization of the imported xylogluco-oligosaccharides.

*R. cellulolyticum*, like most cellulolytic anaerobes, secretes cellulosomes to degrade plant cell walls. They are considered an adaptation to the anaerobic lifestyle, since these complexes are secreted in small amounts and perform better than the cellulolytic systems produced by aerobes composed of free enzymes generally secreted in copious amounts (43–45). The way xyloglucan is processed by *R. cellulolyticum*, which deviates from that reported for the aerobic Gram-negative bacteria Bacteroides ovatus (46) and Cellvibrio japonicus (47), is particularly energy-saving. Our data also demonstrate that the strategy selected by *R. cellulolyticum* permits the utilization of all carbohydrates composing xyloglucan. Thus, this catabolism is likely to represent another efficient adaptation to the anaerobic lifestyle and its ensuing limited energy budget.

## MATERIALS AND METHODS

### Bacterial strains and media.

The strains used in this study are listed in Table S1 in the supplemental material. E. coli strains were grown at 37°C in lysogeny broth supplemented with 100 or 200 μg ml^−1^ ampicillin, 100 μg ml^−1^ chloramphenicol, or 50 μg ml^−1^ kanamycin where applicable. E. coli NEB5α was used for cloning purposes, while E. coli BL21(DE3) was used for protein production.

10.1128/mBio.02206-21.1 TABLE S1 Strains used in this study. Download Table S1, DOCX file, 0.01 MB. Copyright © 2021 Kampik et al. 2021 Kampik et al. https://creativecommons.org/licenses/by/4.0/ This content is distributed under the terms of the Creative Commons Attribution 4.0 International license.

*R*. *cellulolyticum* strains were grown anaerobically at 32°C in basal (48) or minimal medium (49) supplemented with (i) 2 g liter^−1^ arabinose, cellobiose, galactose, glucose, mannose, or xylose or 3.5 g liter^−1^ tamarind xyloglucan and (ii) 5 μg ml^−1^ erythromycin or 2.5 μg ml^−1^ thiamphenicol where applicable. Cultures in xyloglucan-based medium were inoculated (1/10) from precultures grown on arabinose. *R*. *cellulolyticum* H10 ATCC 35319 genomic DNA.

### Construction of *R. cellulolyticum* mutant strains.

The genes at loci Ccel_3221, Ccel_3238, Ccel_3429 and Ccel_3431 were knocked out using the ClosTron technology (25), by retargeting the Ll.ltrB intron on vector pMTL007 with the primers 3221-174a-IBS, 3221-174a-EBS1d, 3221-174a-EBS2 (Ccel_3221), 3238-247s-IBS, 3238-247s-EBS1d, 3238-247s-EBS2 (Ccel_3238), 3429-331s-IBS, 3429-331s-EBS1d, 3429-331s-EBS2 (Ccel_3429), 3431-555a-IBS, 3431-555a-EBS1d, 3431-555a-EBS2 (Ccel_3431), and EBS Universal (see Table S2). Wild-type *R*. *cellulolyticum* (H10) was electrotransformed with the resulting plasmids pMTL-3221, pMTL-3238, pMTL-3429, and pMTL-3431, and the resulting mutant strains MTL3221 (see below), MTL3238, MTL3429 (see below), and MTL3431 were selected as previously described (50, 51). The integration of a single intron at the correct target site was verified by PCR with primers up- and downstream from the target site (3221upmutFw/3221downmutRv, 3238upmutFw/3238downmutRv, 3429upmutFw/3429downmutRv, and 3431upmutFw/3431downmutRv, respectively) and with Southern blots as previously described (52). The unsuccessful inactivation of the gene at locus Ccel_1417 was performed analogously with the primers Ccel_1417-253-IBS/Ccel_1417-253-EBS1d/Ccel_1417-253-EBS2, 1417-769s-IBS/1417-769s-EBS1d/1417-769s-EBS2, or Ccel_1417-933-IBS/Ccel_1417-933-EBS1d/Ccel_1417-933-EBS2.

The integration frequency within Ccel_3221 was too low for the isolation of a single colony. As indicated in a study of Myxococcus xanthus (53), the liquid culture obtained after erythromycin selection was subcultured twice with 1 g liter^−1^ 2-deoxy-d-glucose (2dGlc) before plating.

Unlike the other pMTL007 derivatives, pMTL-3429 could not be cured from the xylose isomerase knockout strain. A modified plasmid (pMTL-*tdk*-3429) carrying gene *tdk* encoding a thymidine kinase was therefore constructed in order to select for loss of *tdk* with 5-fluoro-2-deoxyuridine (FUDR), as previously employed in Clostridium thermocellum (54). The *tdk* gene was amplified from Clostridium acetobutylicum ATCC 824 gDNA with the primers BamHI-tdk-up/NarI-tdk-do and cloned at the BamHI/NarI sites in pSOS952 (55). The P_thl_ promoter in this plasmid was exchanged for the synthetic P4 promoter (56) by site-directed mutagenesis with the primers pSOS-P4-up/pSOS-P4-do. *tdk*, together with the upstream P4 promoter and the downstream terminator found in pSOS952, was then amplified from this plasmid using the primer pairs pSOS-tdk-EcoRV-Fw/tdk-BsrGI-Rv and tdk-BsrGI-Fw/pSOS-tdk-EcoRV-Rv. Overlap PCR of these two amplicons with the primers pSOS-tdk-EcoRV-Fw/pSOS-tdk-EcoRV-Rv introduced a silent mutation (C to T) at the sixth base pair of *tdk*, thereby disrupting the BsrGI site. The resulting DNA fragment was digested with EcoRV and ligated with EheI-linearized pMTL-3429. Wild-type *R*. *cellulolyticum* (H10) was transformed with pMTL-*tdk*-3429 as described above. After erythromycin selection, the culture was subcultured five times in the presence of erythromycin and twice in the presence of erythromycin and 10 μg/ml FUDR (Thermo Fisher, Waltham, MA) before plating.

### Complementation of *R. cellulolyticum* knockout mutants.

The knocked-out genes in *R. cellulolyticum* (and downstream genes where applicable) were amplified with the primer pairs SOS3221F/SOS3221R, SOS3238F/SOS3237R, and SOS3429F/SOS3429R and cloned in pSOS956 (22) at the BamHI/EheI sites, downstream of an attenuated P_thl_ promoter. MTL3238 was complemented with the genes at loci Ccel_3238 and Ccel_3237. All knockout mutants listed above were transformed with both the respective plasmid and the empty pSOS956zeroTm vector (22, 57). A different strategy was used for strain MTL3431 in which the entire pMTL007 vector carrying the intron integrated into the targeted gene. MTL3431 was complemented with the genes at loci Ccel_3431, Ccel_3430, and Ccel_3429; amplified using the primer pair SOS3431F/SOS3429R2; and cloned at the BamHI/EheI sites in pSOS955 (18, 57) carrying a tetracycline resistance cassette. Strain MTL3431 was also transformed with the pSOS955-derivative pSOS955zeroTc.

### Cloning of metabolic enzymes encoding genes in an *E. coli* expression vector.

The genes Ccel_3221, Ccel_3238, Ccel_3429, and Ccel_1417 were amplified from *R. cellulolyticum* genomic DNA using the primer pairs (see Table S2) 3321f/3221r, 3238f/3238r, 3429f/3429r, and 1417f/1417r, respectively. The resulting amplicons were digested by NcoI/XhoI and cloned in NcoI-XhoI-linearized pET28a (Novagen, Madison, WI), thereby leading to pET28a-3221, pET28a-3238, pET28a-3439, and pET28a-1417, which encode C-terminal His-tagged hexokinase, galactokinase, xylose isomerase, and phosphoglucomutase, respectively. After transformation of the E. coli strain NEB5α, positive clones were verified by sequencing. The plasmids were subsequently transferred into the production strain E. coli BL21(DE3).

### Production in *E. coli* and purification of the selected metabolic enzymes.

The transformed E. coli BL21(DE3) strains were grown in Luria-Bertani medium (2 × 700 ml) supplemented with glycerol (12 g liter^−1^) and kanamycin (50 μg/ml) at 37°C until it reached an *A*_600_ of ∼1.5. Induction of the expression was performed overnight at 22°C using 200 μM IPTG (isopropyl-β-d-thiogalactopyranoside). Cells were harvested by centrifugation (3,000 × *g*, 10 min, 4°C), resuspended in 50 ml of 30 mM Tris-HCl (pH 8.0; THC)–5 mM imidazole supplemented with few milligrams of DNase I (Roche, Basel, Switzerland), and broken in a French press (Stansted Fluid Power, Ltd., Harlow, UK). The extract was centrifuged (15,000 × *g*, 10 min 4°C), and the supernatant was loaded on 2 to 4 ml of HisPur Ni-NTA resin (Thermo Scientific, Rockford, IL) equilibrated in the same buffer. Elution of His-tagged proteins of interest was performed using 100 mM imidazole in THC. The purification was achieved on Q-Sepharose fast flow (GE Healthcare, Pittsburgh, PA) equilibrated in THC. Elution was performed using a linear gradient of 0 to 500 mM NaCl in THC. The purified proteins were dialyzed, concentrated by ultrafiltration in a Vivaspin20 (cutoff, 10 kDa; Sartorius, Göttingen, Germany) against 10 mM Tris-HCl (pH 8.0), and stored at −80°C. The concentration of the proteins was estimated based on the absorbance at 280 nm using the program ProtParam tool (www.expasy.org/tools/protparam.html). Purification of CbpA was performed as formerly described (22).

### Characterization of the selected enzymes.

The hexokinase (10 nM) was assayed at 37°C on variable glucose concentrations (0.05 to 5 mM) in 25 mM HEPES (pH 7.0), 25 mM ATP or 2 mM GTP, and 25 mM MgCl_2_. Samples were pipetted at 5 min and diluted in 0.1 M NaOH to stop the reaction prior to analyses by high-pressure anion-exchange chromatography coupled with pulsed amperometric detection (HPAEC-PAD; Thermo Fisher) using a PA1 column (Thermo Fisher) as formerly described (17) or by high-pressure liquid chromatography coupled with a refractive index detector (HPLC-RI). Injection of samples containing glucose and Glu6P or GDP (Sigma, St. Louis, MO) at known concentrations was used for quantification. The hexokinase was assayed on mannose similarly, with variable mannose concentrations (0.05 to 5 mM). Injections of mannose and mannose 6-P (Sigma) and GDP at known concentrations served to quantify the activity. The kinetic parameters of the hexokinase for the NTP were determined by high-pressure liquid chromatography on an Aminex HPX87H column (300 × 7.8 mm; Bio-Rad, Hercules, CA) coupled with a refractive index detector (Iota, Marseille, France): the enzyme (50 nM) was mixed with ATP concentrations varying from 0.67 to 20 mM or with GTP concentrations ranging from 0.1 to 2.5 mM in 25 mM HEPES (pH 7.0), 5 mM glucose, and 25 mM MgCl_2_. Samples (200 μl) were pipetted at 5 and 15 min and mixed with 50 μl of 0.25 M H_2_SO_4_. Portions (25 μl) of diluted samples were analyzed by HPLC-RI (the flow rate was 0.6 ml/min and 55°C): sugars, ATP, and GDP were eluted using 5 mM H_2_SO_4_ for 15 min. Injections of glucose, ATP, and GDP at known concentrations were used to determine the activity. The activity of the hexokinase (25 nM) on 20 mM glucose in 25 mM HEPES (pH 7.0), 5 mM GTP, and 25 mM MgCl_2_ was also assayed in the presence of 20 mM either cellobiose, galactose, or xylose. Then, 100-μl samples were pipetted at 5 min and mixed with 25 μl of 0.25 M H_2_SO_4_ prior to HPLC-RI analyses. The ability to catalyze the reverse reaction was investigated by mixing the hexokinase (13.8 nM) with 1 mM Glu6P in 25 mM HEPES (pH 7.0)–5 mM MgCl_2_, with or without 2 mM GDP, in a final volume of 150 μl. Aliquots (20 μl) were pipetted at 15, 60, 240, and 1440 min and mixed with 180 μl of distilled water and 50 μl of 0.5 M NaOH prior to HPAEC-PAD analyses. In addition, GTP was added in some experiments at concentrations ranging from 0.1 to 2 mM.

The galactokinase (0.5 μM) was incubated at 37°C with variable galactose concentrations (1 to 100 mM) in 25 mM HEPES (pH 7.0), 25 mM ATP or 5 mM GTP, and 25 mM MgCl_2_. Samples (50 to 200 μl) were pipetted at 5 min and diluted in 50 mM H_2_SO_4_ in a final volume of 250 μl. Then, 25-μl samples were subjected to HPLC-RI. Injections of galactose, ATP, and GDP at known concentrations were used to determine the activity. The kinetic parameters of the galactokinase toward NTP were determined as described above using variable ATP concentrations (1 to 25 mM) or variable GTP concentrations (0.25 to 10 mM) in HEPES buffer containing 100 mM galactose and 25 mM MgCl_2_. The activity of the galactokinase was also assayed on 25 mM galactose and 5 mM GTP in the presence of 20 mM either glucose, cellobiose, or xylose. Next, 100-μl samples were pipetted at 5 and 10 min, mixed with 25 μl of 0.25 M H_2_SO_4_, and analyzed by HPLC-RI.

The xylose isomerase (5 μM) was assayed on 10 mM xylose at 37°C in 25 mM HEPES (pH 7.0) buffer containing either 10 mM EDTA, 10 mM MgCl_2_, 10 mM CaCl_2_, 10 mM MnCl_2_, 10 mM CoCl_2_, or no additive. After 15 min of incubation, 100-μl samples were pipetted and mixed with 25 μl of 25 mM H_2_SO_4_, and then 25-μl portions were subjected to HPLC-RI. Injections of xylose and xylulose (Sigma) at known concentrations served to quantify the pentoses. The kinetic parameters on xylose were determined at 37°C by incubating the enzyme (2.5 μM) with variable concentrations of xylose (1 to 100 mM) in HEPES buffer containing 10 mM MgCl_2_. The kinetic parameters of the enzyme on xylulose were established similarly using variable concentrations of xylulose (0.5 to 30 mM). After 10 min of incubation, samples were pipetted, mixed with H_2_SO_4_, and analyzed by HPLC-RI. The xylose isomerase (2.5 μM) on 20 mM xylose was also assayed at 37°C in the same buffer supplemented with either 20 mM glucose, 20 mM galactose, or 20 mM cellobiose. After 10 min of incubation, 100-μl samples were pipetted and mixed with 25 μl of 25 mM H_2_SO_4_ prior to analyses, as described above.

The cellobiose phosphorylase CbpA at 0.5 μM on 10 mM cellobiose in 50 mM phosphate buffer was assayed as formerly reported (22) but also in the presence of either 20 mM galactose, 20 mM xylose, or 20 mM glucose. For glucose, determination of the *K_i_* value was established by incubating the enzyme (0.5 μM) at 37°C with variable cellobiose concentrations (0.05 to 10 mM) in the same buffer supplemented with glucose at 0, 1, or 5 mM. Samples (10 to 100 μl) were pipetted at 2 and 5 min of incubation, diluted in NaOH at a final concentration of 0.1 M, and analyzed by HPAEC-PAD.

The activity of the α-phosphoglucomutase on Glu1P was determined by incubating the enzyme (50 nM) at 37°C with Glu1P at concentrations ranging from 50 μM to 2 mM, in 25 mM HEPES (pH 7.0) buffer. Aliquots (10 to 200 μl) were pipetted at 1.5 or 2.5 min and diluted in NaOH at a final concentration of 0.1 M. Next, 25-μl portions of diluted samples were subsequently analyzed by HPAEC-PAD. The kinetic parameters of the enzyme on Glu6P were established similarly except that the incubation time was 5 min and the concentration of Glu6P ranged from 50 μM to 5 mM. Injections of samples containing known concentrations of Glu1P and Glu6P were used to quantify the phosphorylated hexoses.

### Preparation of *R. cellulolyticum* crude extract and determination of the concentration of selected metabolic enzymes.

Cells were grown in xyloglucan-based medium (120 ml) until the optical density at 450 nm (OD_450_) reached 0.8. The culture was centrifuged (10,000 × *g*, 10 min, 4°C), and the pellet was resuspended in 8 ml of 25 mM sodium phosphate buffer (pH 7.0) containing 50 mM NaCl. The suspension was broken in a French press and centrifuged (20,000 × *g*, 10 min, 4°C). The supernatant was concentrated to 600 μl on a Vivaspin20 (Sartorius; cutoff, 10 kDa), aliquoted, and kept at −80°C. The CbpA concentration in the crude extract was estimated by incubating various volumes of the crude extract (1.5 to 4 μl) or various concentrations of the purified CbpA (0.1 to 0.5 μM) with 5 mM cellobiose in 50 mM phosphate buffer (pH 7.0). The consumption of cellobiose was followed by HPLC-RI. The hexokinase concentration was determined by incubation of various volumes of the crude extract (1.5 to 4 μl) or various concentrations of purified hexokinase (2.5 to 7.5 nM) with 5 mM glucose in 25 mM HEPES (pH 7.0) buffer containing 2 mM GTP and 25 mM MgCl_2_. Glucose consumption was monitored by HPLC-RI detection. The concentration of xylose isomerase was estimated by incubating various volumes of the crude extract (2.5 to 7 μl) or various concentrations of the purified xylose isomerase (1 to 2.5 μM) with 5 mM xylose in HEPES buffer containing 10 mM MgCl_2_. The consumed xylose was monitored by HPLC-RI. The galactokinase concentration was estimated by incubation of various volumes of the crude extract (2.5 to 7 μl) or various concentrations of the purified galactokinase (50 to 200 nM) with 5 mM galactose in HEPES buffer containing 5 mM GTP and 25 mM MgCl_2_. The consumed galactose was monitored by HPLC-RI. The α-phosphoglucomutase concentration was estimated as follows. Various volumes (2 to 7 μl) of the crude extract or various concentrations of purified phosphoglucomutase (5 to 10 nM) were incubated with 1 mM Glu1P in HEPES buffer. The consumption of the Glu1P was then followed by HPAEC-PAD, as described above.

### Activity of combined purified enzymes and crude extract on mixtures of simple sugars.

The purified enzymes were mixed according to their proportions in the crude extract. A solution containing 20 μM xylose isomerase, 3.42 μM CbpA, 0.61 μM galactokinase, 0.277 μM hexokinase, and 0.137 μM α-phosphoglucomutase in 10 mM HEPES (pH 7.0) buffer was prepared. First, 50 μl of purified enzyme mix (or 50 μl of 10 mM HEPES buffer) was incubated at 37°C with 950 μl of 25 mM HEPES (pH 7.0) buffer containing 2 mM ATP or 2 mM GTP, 5 mM MgCl_2_, 10 mM phosphate, 0.4 mM cellobiose, 0.8 mM glucose, 1.2 mM xylose, and 0, 0.4, or 0.8 mM galactose. At 0 min, 15 min, 1 h, 4 h, and 24 h, 20-μl samples were pipetted and mixed with 180 μl of distilled water and 50 μl of 50 mM or 0.1 M NaOH for HPAEC-PAD analyses, whereas 120 μl of the sample was mixed with 30 μl of 0.25 M H_2_SO_4_ for analyses by HPLC-RI. For HPAEC-PAD analyses, 25-μl portions of diluted samples were applied on a PA20 column (150 × 3 mm; Thermo Fisher) with the corresponding guard column (30 × 3 mm) at 35°C. Sugars were eluted with the buffers 0.1 M NaOH, 0.1 M NaOH + 1 M sodium acetate, and distilled water as the eluents A, B, and C, respectively, using the following multistep procedure: isocratic separation (10 min, 27% A + 73% C), separation gradient (20 min, 2% B + 98% C to 19% B + 81% C), column wash (5 min, 100% A), and subsequent column equilibration (10 min, 27% A + 73% C). The flow was at 0.45 ml/min. HPLC-RI analyses and HPAEC-PAD using a PA1 column were also performed as described above. Injections of galactose, glucose, xylose, cellobiose, Gal1P, Glu1P, and Glu6P (supplemented with xylulose for HPLC-RI analyses) at known concentrations served to quantify the various sugars.

For the crude extract, the activity was monitored similarly, but only HPAEC-PAD analyses using PA20/PA1 columns were performed. For the experiment with a pulse of 2 mM GTP (or buffer) after 1 h (sugar mix 1), the consumption of the various sugars was monitored by HPAEC-PAD using a PA1 column as described above.

### Analyses of culture supernatants.

For growth on xyloglucan, 200-μl samples of the cultures were taken at the midexponential and late exponential phases of growth and centrifuged for 10 min (15,000 × *g*, 4°C). Next, 20-μl portions of the supernatants were diluted in 250 μl (final volume) of 0.1 M NaOH and analyzed by HPAEC-PAD using PA1 or PA20 columns, as described above. Injection of samples containing glucose, galactose, xylose, cellobiose, and XGO_4_ (9) (Megazyme, Bray, Ireland) at known concentrations was used to quantify the sugars.

### Inhibition assays of xylogluco-oligosaccharide depolymerizing enzymes.

Gal42A (2 nM) was first assayed at 37°C on 2 mM *p*-nitrophenyl-β-d-galactoside (*p*NPβGal) at 2 mM in 50 mM phosphate (pH 7.0) buffer as formerly reported (9) with or without 20 mM either galactose, glucose, xylose, or cellobiose. The released *p*NP was monitored at 400 nm for 250 s. To determine the *K_i_* value for galactose, Gal42A (2 nM) was assayed again on *p*NPβGal at variable concentrations (0.5 to 7 mM) in the presence of either galactose (at 0, 2, or 10 mM) or cellobiose (at 0, 4, or 20 mM). Glu3A (20 nM) was initially assayed at 37°C on 5 mM *p*-nitrophenyl-β-d-glucoside (9) (*p*NPβGlu) in 50 mM phosphate (pH 7.0) buffer in the absence or presence of 20 mM galactose, 20 mM glucose, 20 mM xylose, or 20 mM cellobiose, as described above. Since significant inhibition was only observed for glucose and cellobiose, their *K_i_* values were determined by performing new kinetics studies on *p*NPβGlu (at concentrations ranging from 1 to 16 mM) in the presence of 0, 2, or 10 mM glucose or 0, 10, or 30 mM cellobiose.

Xyl31A (100 nM) was first assayed at 37°C on 1 mM isoprimeverose (Megazyme) as formerly reported (9) in 50 mM phosphate (pH 6.0) in the absence or presence of 10 or 50 mM xylose, 10 mM galactose, 10 mM glucose, or 10 mM cellobiose. After 5 and 20 min of incubation, 20-μl portions were pipetted and mixed with 180 μl of distilled water and 50 μl of 0.5 M NaOH prior to analyses by HPAEC-PAD using a PA1 column. The *K_i_* for glucose was estimated by performing new kinetics analyses using variable isoprimeverose concentrations (0.5 to 8 mM) in the presence of 0, 2, or 10 mM glucose.
