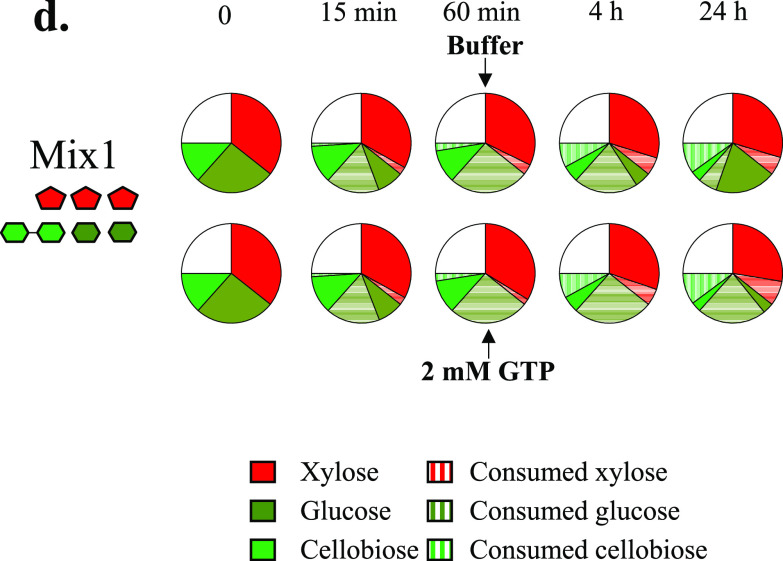# Erratum for Kampik et al., “Handling Several Sugars at a Time: a Case Study of Xyloglucan Utilization by *Ruminiclostridium cellulolyticum*”

**DOI:** 10.1128/mbio.03551-21

**Published:** 2022-01-11

**Authors:** Clara Kampik, Nian Liu, Mohamed Mroueh, Nathalie Franche, Romain Borne, Yann Denis, Séverine Gagnot, Chantal Tardif, Sandrine Pagès, Stéphanie Perret, Nicolas Vita, Pascale de Philip, Henri-Pierre Fierobe

**Affiliations:** a Aix-Marseille Université, CNRS, LCB-UMR7283, Marseille, France

## ERRATUM

Volume 12, no. 6, e02206-21, 2021, https://doi.org/10.1128/mBio.02206-21. The first error regards Table 3, which was published with a wrong title. The correct title is “*K_i_* concn (mM) ± SD^a^.” Second, in the originally published [Fig fig1]Fig. 5d, the proportions of consumed glucose, xylose, and cellobiose cannot be distinguished from the proportions of glucose, xylose, and cellobiose which have not been processed. The corrected Fig. 5d, with improved colors to facilitate the visualization, appears below. There are no corresponding changes to the text of the figure legend.

**Figure fig1:**