# Supplementary material for: Handling Several Sugars at a Time: a Case Study of Xyloglucan Utilization by Ruminiclostridium cellulolyticum
Source: mBio. 2021 Nov 9;12(6):e02206-21. doi: 10.1128/mBio.02206-21 (PMC8576529; doi:10.1128/mBio.02206-21)
Supplement: FIG S1 [file mbio.02206-21-sf001.docx]

Figure S1: Relative expression of genes encoding the selected metabolic enzymes in *R. cellulolyticum*.

The cultures used for RNA preparation were grown on arabinose (white) or xyloglucan (blue). They were inoculated from arabinose-containing precultures. For each gene, the relative expression on a given growth substrate is standardized with 16S rRNA encoding gene amplification and given versus arabinose. Error bars show the standard error of three biological replicates. RNA extraction, reverse transcription and quantitative real-time PCR (qPCR) analyses were performed as formerly described (Kampik *et al*., 2020). Briefly, total RNAs were prepared from *R. cellulolyticum* 20-ml cultures grown until late exponential phase, and the RNA was quantified and reverse-transcribed. Quantitative real-time PCR (RT-qPCR) analyses were performed on a CFX96 Real-Time System (Bio-Rad) and the results were analyzed using Bio-Rad CFX Maestro software, version 1.1 (Bio-Rad, France) (Kampik *et al*., 2020). The RNA16S gene was used as a reference for normalization. Primer pairs are reported in Table S2. Gene loci and corresponding enzymes are given below each graph.

Reference

Kampik C, Denis Y, Pagès S, Perret S, Tardif C, Fierobe H-P, de Philip P. 2020. A novel two-component system, XygS/XygR, positively regulates xyloglucan degradation, import, and catabolism in *Ruminiclostridium cellulolyticum*. *Appl Environ Microbiol* 86:e01357-20.
