# Supplementary material for: Handling Several Sugars at a Time: a Case Study of Xyloglucan Utilization by Ruminiclostridium cellulolyticum
Source: mBio. 2021 Nov 9;12(6):e02206-21. doi: 10.1128/mBio.02206-21 (PMC8576529; doi:10.1128/mBio.02206-21)
Supplement: TABLE S2 [file mbio.02206-21-st002.docx]

Table S2 : list of primers used

| Name | Sequence (5’→3’) | | | Target |
| --- | --- | --- | --- | --- |
| **Primers used for cloning of the targeted genes in *E. coli* expression vector pET28a** | | | | |
|  | | | | |
| Pgm1417NcoF | | GGGGGGCCATGGAATACCTATCAAAACTTAACTT | Ccel_1417 | |
| Pgm1417XhoR | | TTTTTTCTCGAGTAAGAGTGGCTTTATAACATCAAGAACG | Ccel_1417 | |
| HK3221NcoF | | AAAAAACCATGGGGTCCAAATTAGAAATAGTACAGG | Ccel_3221 | |
| HK3221XhoR | | AAAAAACTCGAGTTGTGTTACGGCAAACGCAGCAGCTATTG | Ccel_3221 | |
| Gal3238NcoF | | GGGGTTCCATGGTGCAGAAAAATTATGATGAGCTAAAG | Ccel_3238 | |
| Gal3238XhoR | | TTTTTTCTCGAGAATCTTGATTTCCCTTGCTCCGTCACT | Ccel_3238 | |
| Xi3429NcoF | | AGGGGGCCATGGTGTCAGAAGTATTTAGCGGTATTTCAAAC | Ccel_3429 | |
| Xi3429XhoR | | TTTTTTCTCGAGTTTTGTTTCTAGGATATACTGGTTTAACAAAG | Ccel_3429 | |
|  |  | | |  |
| **Primers used for construction of *R. cellulolyticum* mutant strains** | | | | |
|  |  | | |  |
| 1417-769S-IBS | | AAAAAAGCTTATAATTATCCTTAGGCTTCAAGGAAGTGCGCCCAGATAGGGTG | pMTL007 / Ccel_1417 | |
| 1417-769S-EBS1d | | CAGATTGTACAAATGTGGTGATAACAGATAAGTCAAGGAAGTTAACTTACCTTTCTTTGT | pMTL007 / Ccel_1417 | |
| 1417-796S-EBS2 | | TGAACGCAAGTTTCTAATTTCGATTAAGCCTCGATAGAGGAAAGTGTCT | pMTL007 / Ccel_1417 | |
| 1417-253S-IBS | | AAAAAAGCTTATAATTATCCTTACTATTCCAATCCGTGCGCCCAGATAGGGTG | pMTL007 / Ccel_1417 | |
| 1417-253S-EBS1d | | AGATTGTACAAATGTGGTGATAACAGATAAGTCCAATCCCTTAACTTACCTTTCTTTGT | pMTL007 / Ccel_1417 | |
| 1417-253S-EBS2 | | TGAACGCAAGTTTCTAATTTCGGTTAATAGTCGATAGAGGAAAGTGTCT | pMTL007 / Ccel_1417 | |
| 1417-933S-IBS | | AAAAAAGCTTATAATTATCCTTAAGAGTCGGCGTGGTGCGCCCAGATAGGGTG | pMTL007 / Ccel_1417 | |
| 1417-933S-EBS1d | | AGATTGTACAAATGTGGTGATAACAGATAAGTCGGCGTGGTTAACTTACCTTTCTTTGT | pMTL007 / Ccel_1417 | |
| 1417-933S-EBS2 | | TGAACGCAAGTTTCTAATTTCGATTACTCTTCGATAGAGGAAAGTGTCT | pMTL007 / Ccel_1417 | |
| 3221-174a-EBS1d | | CAGATTGTACAAATGTGGTGATAACAGATAAGTCACCCTGTTTAACTTACCTTTCTTTGT | pMTL007 / Ccel_3221 | |
| 3221-174a-EBS2 | | TGAACGCAAGTTTCTAATTTCGGTTCTTTTCCGATAGAGGAAAGTGTCT | pMTL007 / Ccel_3221 | |
| 3238-247s-IBS | | AAAAAAGCTTATAATTATCCTTAGAGGTCGAACTGGTGCGCCCAGATAGGGTG | pMTL007 / Ccel_3238 | |
| 3238-247s-EBS1d | | CAGATTGTACAAATGTGGTGATAACAGATAAGTCGAACTGGATAACTTACCTTTCTTTGT | pMTL007 / Ccel_3238 | |
| 3238-247s-EBS2 | | TGAACGCAAGTTTCTAATTTCGATTACCTCTCGATAGAGGAAAGTGTCT | pMTL007 / Ccel_3238 | |
| 3429-331s-IBS | | AAAAAAGCTTATAATTATCCTTACCTGACGGCGACGTGCGCCCAGATAGGGTG | pMTL007 / Ccel_3429 | |
| 3429-331s-EBS1d | | CAGATTGTACAAATGTGGTGATAACAGATAAGTCGGCGACACTAACTTACCTTTCTTTG | pMTL007 / Ccel_3429 | |
| 3429-331s-EBS2 | | TGAACGCAAGTTTCTAATTTCGGTTTCAGGTCGATAGAGGAAAGTGTCT | pMTL007 / Ccel_3429 | |
| 3431-555a-IBS | | AAAAAAGCTTATAATTATCCTTACTCTCCTTTAGCGTGCGCCCAGATAGGGTG | pMTL007 / Ccel_3431 | |
| 3431-555a-EBS1d | | CAGATTGTACAAATGTGGTGATAACAGATAAGTCTTTAGCAATAACTTACCTTTCTTTGT | pMTL007 / Ccel_3431 | |
| 3431-555a-EBS2 | | TGAACGCAAGTTTCTAATTTCGGTTGAGAGTCGATAGAGGAAAGTGTCT | pMTL007 / Ccel_3431 | |
| EBS universal primer | | CGAAATTAGAAACTTGCGTTCAGTAAAC | pMTL007 | |
| BamHI-tdk-up | | GGGGGGGATCCAGAATTTAAAAGGAGGGATTAAAATGTACAGACCAAAGGACCAC | C_AC2887 | |
| NarI-tdk-do | | GGGGGGGCGCCTTACACCTCTTTTTTTTGTGGAAC | C_AC2887 | |
| pSOS-P4-up | | GTTAGCCAAAGCTCCTGCAGGTCGATCTAGATAGACATAATATATTGACAAATTTATTTTTTAAAGTTA |  | |
| pSOS-P4-do | | CATTTTAATCCCTCCTTTTAAATTCTGGATCCCAACTTAATTTTAACTTTAAAAAATAAATTTGTC |  | |
| pSOS-tdk-EcoRV-Fw | | ACTCAGATATCGGAATGGCGTGTGTGTTAG | pSOS952 / C_AC2887 | |
| pSOS-tdk-EcoRV-Rv | | ACTCAGATATCAACAGCTATGACCATGATTACG | pSOS952 / C_AC2887 | |
| tdk-BsrGI-Fw | | AATGTATAGACCAAAGGACCACGGATG | C_AC2887 | |
| tdk-BsrGI-Rv | | CGTGGTCCTTTGGTCTATACATTTTAATCCCTCC | C_AC2887 | |
|  | |  |  | |
| **Primers used for cloning in pSOS956 or pSOS955 *R. cellulolyticum* expression vectors** | | | | |
|  | | | | |
| SOS3221F | | CTAGGATCCAGAATTTAAAAGGAGGGATTAAAATGGGGTCCAAATTAGAAATAGTACAG | Ccel_3221 | |
| SOS3221R | | CATAGTGGCGCCCTATTGTGTTACTGCAAACGCAGCAGC | Ccel_3221 | |
| SOS3238F | | CTAGGATCCAGAATTTAAAAGGAGGGATTAAAATGCAGAAAAATTATGATGAGCTAAAG | Ccel_3238 | |
| SOS3237R | | CATAGTGGCGCCTCACCCGTTATACCCGTTTGG | Ccel_3237 | |
| SOS3429F | | CTAGGATCCAGAATTTAAAAGGAGGGATTAAAATGTCAGAAGTATTTAGCGGTATTTCAAAC | Ccel_3429 | |
| SOS3429R | | CATAGTGGCGCCTTATTTTGTTTCGAGGATATACTGGTTTAACAAAG | Ccel_3429 | |
| SOS3431R | | AGGATCCAGAATTTAAAAGGAGGGATTAAAATGTCTTTTCTTATTGGTATTGATCTAGGAAC | Ccel_3431 | |
| SOS3429R-2 | | CTCTTTTACTGGCAAATCATTAAGTGGCGCCTTATTTTGTTTCGAGGATATACTGGTTTAAC | Ccel_3429 | |
|  | |  |  | |
| **Primers used for RT-qPCR experiments** | | | | |
|  | | | | |
| 1417F | | AGAGTTCTCACTGGAGGCTG | Ccel_1417 | |
| 1417R | | CTTGCAGCGTCTTCCTTTGA | Ccel_1417 | |
| 2109F | | ATGAGTGCAGGCATGGTTTG | Ccel_2109 | |
| 2109R | | ACCGAGTTCTCAACTTCAACC | Ccel_2109 | |
| 3221F | | TGGCTTCGGATACCCTCTTT | Ccel_3221 | |
| 3221R | | AGTCAAATACCGGGAGCACA | Ccel_3221 | |
| 3237F | | GTGATTCCGGAGAATGAGCC | Ccel_3237 | |
| 3237R | | GCAAGAAGCCGGAGTAAACA | Ccel_3237 | |
| 3238F | | GCCTTCTGCACATTCTCTGA | Ccel_3238 | |
| 3238R | | TGGCTGTACTGGGACAAAGA | Ccel_3238 | |
| 3429F | | GTTGGAATGTGTGTCCTGCT | Ccel_3429 | |
| 3429R | | CTGGGGTGGTAGAGAAGGTT | Ccel_3429 | |
|  | |  |  | |
| **Primers used for PCR analyses and sequencing** | | | | |
|  | |  |  | |
| T7 | | TAATACGACTCACTATAGGG | pET28a | |
| T7term | | GCTAGTTATTGCTCAGCGG | pET28a | |
| avalfac | | TAAGGAGGTGTATTTCATATGAC | pMTL007 | |
| SOSdir | | ACTATTGGTTGGAATGGCGTG | pSOS95 | |
| NarRev | | AGCTCTAGGCAATATTATATCTGCAAGAATG | pSOS95 | |
| 1417-831F | | TCCCGAAGAAAGATCCGCCT | Ccel_1417 | |
| 3221-666F | | GAACAATCATCCTCTTAATGGTC | Ccel_3221 | |
| 3221upmutFw | | GTATGCTGCGTACTGCAATG | Ccel_3221 | |
| 3221downmutRv | | ATTCTTCCGGCTCTAGCAAC | Ccel_3221 | |
| 3238-511F | | GAATTCTGCGGAGTAAGCTG | Ccel_3238 /Ccel_3237 | |
| 3238upmutFw | | CCCGGAAGAGTAAATCTCATCG | Ccel_3238 | |
| 3238downmutRv | | TGCCGCAGAAGATGAAAGTC | Ccel_3238 | |
| 3429-518F | | GCAGCTCAGGTTAAAAAGGC | Ccel_3429 | |
| 3429-708F | | AGAACCAACTAAGCACCAATATG | Ccel_3429 | |
| 3429upmutFw | | GCAGTTATCGGCGGAAAG | Ccel_3429 | |
| 3429downmutRv | | ACCTGAGCTGCTGCATAC | Ccel_3429 | |
| 3430-385F | | GTCCATAAGGCTTATGGAAG | Ccel_3430 | |
| 3430-1086F | | GTATTGCATCTGCCAAGGCA | Ccel_3430 | |
| 3431-1177F | | GGGTGTTACTTTCAGTTTGAGGG | Ccel_3431 | |
| 3431-644R | | CTGAAGAAGTAACCTTTCCGG | Ccel_3431 | |
| 3431upmutFw | | GCAAACCCGGCACTTACAGGATTTAC | Ccel_3431 | |
| 3431downmutRv | | TGCTGCCTGATCTCCCCCACC | Ccel_3431 | |
