# Supplementary material for: Handling Several Sugars at a Time: a Case Study of Xyloglucan Utilization by Ruminiclostridium cellulolyticum
Source: mBio. 2021 Nov 9;12(6):e02206-21. doi: 10.1128/mBio.02206-21 (PMC8576529; doi:10.1128/mBio.02206-21)
Supplement: FIG S2 [file mbio.02206-21-sf002.docx]

Figure S2: Non-linear (Michaelis-Menten) regression analysis of the activities of the selected metabolic enzymes.

a): analysis of the phosphorylating activity of the hexokinase on glucose in presence of 25 mM ATP (black squares and line) or 2 mM GTP (red circles and line). b): analysis of the hydrolytic activity of the hexokinase on ATP in presence of 5 mM glucose. c): analysis of the hydrolytic activity of the hexokinase on GTP in presence of 5 mM glucose. d): analysis of the phosphorylating activity of the hexokinase on mannose in presence of 25 mM ATP (black squares and line) or 2 mM GTP (red circles and line).

e): analysis of the activity of cellobiose phosphorylase on cellobiose in presence of 0, 1 or 5 mM glucose.

f): analysis of the phosphorylating activity of the galactokinase on galactose in presence of 25 mM ATP (black squares and line) or 5 mM GTP (red circles and line). g): analysis of the phosphorylating activity of the galactokinase in presence of 100 mM galactose and variable concentrations of ATP. h): analysis of the phosphorylating activity of the galactokinase in presence of 100 mM galactose and variable concentrations of GTP.

i): analysis of the activity of the xylose isomerase on xylose. j): analysis of the activity of the xylose isomerase on xylulose.

k): analysis of the activity of the α-phosphoglucomutase using Glu1P as the substrate. l): analysis of the activity of the α-phosphoglucomutase using Glu6P as the substrate.

The activities were monitored by HPAEC-PAD, using a PA1 column (a (ATP); d (ATP); e, k and l) or using a refractive index detector and an Aminex HPX87H column (a (GTP), b, c, d (GTP), f, g, h, i and j). The data show the mean of two to four independent experiments and bars indicate the standard deviations. Curves fitting was performed using the Origin 2019b software.
