# Supplementary material for: Handling Several Sugars at a Time: a Case Study of Xyloglucan Utilization by Ruminiclostridium cellulolyticum
Source: mBio. 2021 Nov 9;12(6):e02206-21. doi: 10.1128/mBio.02206-21 (PMC8576529; doi:10.1128/mBio.02206-21)
Supplement: FIG S3 [file mbio.02206-21-sf003.docx]

Figure S3: Southern blot analyses of the various mutant strains.

Genomic DNA of the wild-type and mutant strains, and plasmidic DNA of pMTL-3431 were digested with the restriction enzymes indicated above the lanes and submitted to gel electrophoresis and transfer as previously described (Kampik *et al*., 2020). The blot was then submitted to hybridization using a labeled probe targeting the erythromycin cassette present in the intron.

(A) Analysis of mutant strains MTL3238 and MTL3221. The presence of single bands at the expected sizes 3.7 bp (EcoRI), 6.7 bp (EcoRV) and 3.0 bp (HindIII) for MTL3238 and at 4.6 bp (EcoRI), 6.6 bp (EcoRV) and 2.8 bp (HindIII) for MTL3221 demonstrated single intron integrations in the respective target genes.

(B) Analysis of mutant strain MTL3429. The presence of single bands at the expected sizes 3.6 bp (EcoRV) and 4.4 bp (HindIII) demonstrated a single intron integration in the target gene. The size of the observed band for the restriction enzyme HaeIII indicated that the downstream HaeIII site closest to the intron in this strain was not recognized, leading to a DNA fragment of 4.1 bp (between the closest upstream HaeIII site and the next downstream HaeIII site) instead of 2.9 bp.

(C) Analysis of mutant strain MTL3431. The size of the observed bands indicated that the gene at locus Ccel_3431 had been interrupted by the single integration of plasmid pMTL-3431 used for the strain construction within the target gene, as illustrated in (D).

(D) Schematic representation of genetic regions of interest in relation to the construction of the xylulokinase knockout mutant MTL3431. Genes are shown in black, introns of the Clostron technology in white, and the backbone of the plasmid pMTL-3431 in gray. The latter is not drawn proportional in size, as indicated by dashed gray lines. Restriction sites relevant for DNA fragments after hybridization with the probe are indicated for pMTL-3431 (a), the result of the intron insertion without recombination (b) and the result of the vector integration after a recombination event, which can lead to two schemes (c and d). The DNA fragments obtained after hybridization are represented as dashed lines, and their size is given in kb in brackets below the lines. The results indicated that the xylulokinase mutant strain corresponds to the genotype with a plasmid integration as shown in (d).

Reference

Kampik C, Denis Y, Pagès S, Perret S, Tardif C, Fierobe H-P, de Philip P. 2020. A novel two-component system, XygS/XygR, positively regulates xyloglucan degradation, import, and catabolism in *Ruminiclostridium cellulolyticum*. *Appl Environ Microbiol* 86:e01357-20.
