# Supplementary material for: Handling Several Sugars at a Time: a Case Study of Xyloglucan Utilization by Ruminiclostridium cellulolyticum
Source: mBio. 2021 Nov 9;12(6):e02206-21. doi: 10.1128/mBio.02206-21 (PMC8576529; doi:10.1128/mBio.02206-21)
Supplement: FIG S5 [file mbio.02206-21-sf005.docx]

Figure S5: Analyses of the culture supernatants of a) wild-type and b) MTL3431 strains grown on xyloglucan-based medium.

Culture supernatants were analyzed by HPAEC-PAD on a PA20 column. Lower curves correspond to mid-exponential phase of growth and upper curves correspond to late exponential phase of growth. The retention times of galactose, glucose and xylose are shown (dotted lines).
