# Supplementary material for: Handling Several Sugars at a Time: a Case Study of Xyloglucan Utilization by Ruminiclostridium cellulolyticum
Source: mBio. 2021 Nov 9;12(6):e02206-21. doi: 10.1128/mBio.02206-21 (PMC8576529; doi:10.1128/mBio.02206-21)
Supplement: FIG S6 [file mbio.02206-21-sf006.docx]

Figure S6: Inhibitions of the three cytoplasmic xyloglucandextrin-depolymerizing enzymes.

a) Inhibition of the β-galactosidase Gal42A by galactose and cellobiose. Activity was monitored spectrophotometrically (A_400_) on the chromogenic substrate *p*-nitrophenyl-β-D-galactoside (*p*NPβGal). b) Inhibition of the β-glucosidase Glu3A by glucose and cellobiose. Activity was monitored spectrophotometrically (A_400_) on the chromogenic substrate *p*-nitrophenyl-β-D-glucoside (*p*NPβGlu). c) Inhibition of the α-xylosidase Xyl31A by glucose. Activity was monitored on the disaccharide isoprimeverose by HPAEC-PAD using a PA1 column. The data show the mean of two independent experiments and bars represent the standard deviations. Curves fitting was performed using the Origin 2019b software.
