# Supplementary material for: Handling Several Sugars at a Time: a Case Study of Xyloglucan Utilization by Ruminiclostridium cellulolyticum
Source: mBio. 2021 Nov 9;12(6):e02206-21. doi: 10.1128/mBio.02206-21 (PMC8576529; doi:10.1128/mBio.02206-21)
Supplement: TABLE S1 [file mbio.02206-21-st001.docx]

Table S1 : Strains used in this study

| Name | Relevant properties | Source/reference |
| --- | --- | --- |
| ***R. cellulolyticum* strains** |  |  |
|  |  |  |
| *R. cellulolyticum* | Wild-type strain | ATCC 35319 |
| *R. cellulolyticum* (p0) | Wild-type strain containing empty pSOS956 (*tm^R^*) | Fosses *et al.*, 2017 |
| *R. cellulolyticum* (p0-tc) | Wild-type strain containing empty pSOS955 (*tet^R^*) | Kampik *et al*, 2020 |
| *R. cellulolyticum* MTL2109 | *ccel_2109* ::int (*erm^R^*) | Liu *et al.,* 2019 |
| *R. cellulolyticum* MTL2109 (p0) | *ccel_2109* ::int (*erm^R^*) containing empty pSOS956 (*tm^R^*) | Liu *et al.,* 2019 |
| *R. cellulolyticum* MTL2109 (p2109) | *ccel_2109* ::int (*erm^R^*) containing pSOS956-2109 (*tm^R^*) | Liu *et al.,* 2019 |
| *R. cellulolyticum* MTL3221 | *ccel_3221* ::int (*erm^R^*) | This study |
| *R. cellulolyticum* MTL3221 (p0) | *ccel_3221* ::int (*erm^R^*) containing empty pSOS956 (*tm^R^*) | This study |
| *R. cellulolyticum* MTL3221 (p3221) | *ccel_3221* ::int (*erm^R^*) containing pSOS956-3221 (*tm^R^*) | This study |
| *R. cellulolyticum* MTL3238 | *ccel_3238* ::int (*erm^R^*) | This study |
| *R. cellulolyticum* MTL3238 (p0) | *ccel_3238* ::int (*erm^R^*) containing empty pSOS956 (*tm^R^*) | This study |
| *R. cellulolyticum* MTL3238 (p3238-3237) | *ccel_3238* ::int (*erm^R^*) containing pSOS956-3238-3237 (*tm^R^*) | This study |
| *R. cellulolyticum* MTL3429 | *ccel_3429* ::int (*erm^R^*) | This study |
| *R. cellulolyticum* MTL3429 (p0) | *ccel_3429* ::int (*erm^R^*) containing empty pSOS956 (*tm^R^*) | This study |
| *R. cellulolyticum* MTL3429 (p3429) | *ccel_3429* ::int (*erm^R^*) containing pSOS956-3429 (*tm^R^*) | This study |
| *R. cellulolyticum* MTL3431 | *ccel_3431* ::int (*erm^R^*) | This study |
| *R. cellulolyticum* MTL3431  (p0) | *ccel_3431* ::int (*erm^R^*) strain containing empty pSOS955 (*tet^R^*) | This study |
| *R. cellulolyticum* MTL3431  (p3431-3430-3429) | *ccel_3431* ::int (*erm^R^*) strain containing pSOS955-3431-3430-3429 (*tet^R^*) | This study |
|  |  |  |
| ***C. acetobutylicum* strain** |  |  |
|  |  |  |
| *C. acetobutylicum* | Wild-type strain | ATCC 824 |
|  |  |  |
